# Thrust-dominated unilateral rupture of a blind listric fault associated with the 2024 Hualien earthquake

**DOI:** 10.1038/s41598-024-82971-x

**Published:** 2024-12-28

**Authors:** Ao Zheng, Xiangwei Yu, Chengyuan Bai, Wenbin Xu, Jiaqi Qian, Wenbo Zhang, Xiaofei Chen

**Affiliations:** 1 College of Earth and Planetary Sciences, University of Chinese Academy of Sciences, Beijing, China; 2 School of Resources and Geosciences, China University of Mining and Technology, Xuzhou, China; 3 School of Geosciences and Info-Physics, Central South University, Changsha, China; 4 Department of Earth and Space Sciences, Southern University of Science and Technology, Shenzhen, China

**Keywords:** Geophysics, Seismology

## Abstract

The 2024 Hualien *M*_w_ 7.4 earthquake struck the Longitudinal Valley, which accommodates the partial collision between the Eurasian and Philippine Sea plates. As the most significant event in Taiwan since the 1999 Chi-Chi *M*_w_ 7.6 earthquake, it presents a distinct opportunity for investigating the current rupture behavior related to the northern Longitudinal Valley. The spatiotemporal rupture process of the Hualien earthquake is reconstructed through the analysis of geodetic and seismic observations. We demonstrate that the Hualien earthquake occurs on a blind listric fault, manifesting as a unilateral rupture primarily extending toward the NNE. The slip distribution exhibits a compact pattern dominated by the thrust faulting. As indicated by the increased Coulomb failure stress, the 2022 Chihshang *M*_w_ 6.5 and *M*_w_ 6.9 earthquakes cause a triggering effect on the Hualien earthquake. The Hualien earthquake also promotes the occurrence of a seismic swarm at the southernmost tip of its rupture area.

**Supplementary Information:**

The online version contains supplementary material available at 10.1038/s41598-024-82971-x.

## Introduction

On 2 April 2024, an earthquake of *M*_w_ 7.4 struck Hualien, the largest coastal city in eastern Taiwan. The hypocenter of the earthquake located by the U.S. Geological Survey-National Earthquake Information Center (USGS-NEIC) is offshore at 23.835°N, 121.598°E, with a depth of 40.0 km. This earthquake caused intense shaking throughout the entire island of Taiwan, resulting in a maximum intensity of up to 6+ (440–800 cm/s^2^) in the NE of Hualien according to the modified seismic intensity scale of Taiwan. Unfortunately, the earthquake caused extremely severe damages and losses, leading to at least 18 casualties and more than 1,100 injuries.

**Fig. 1:**
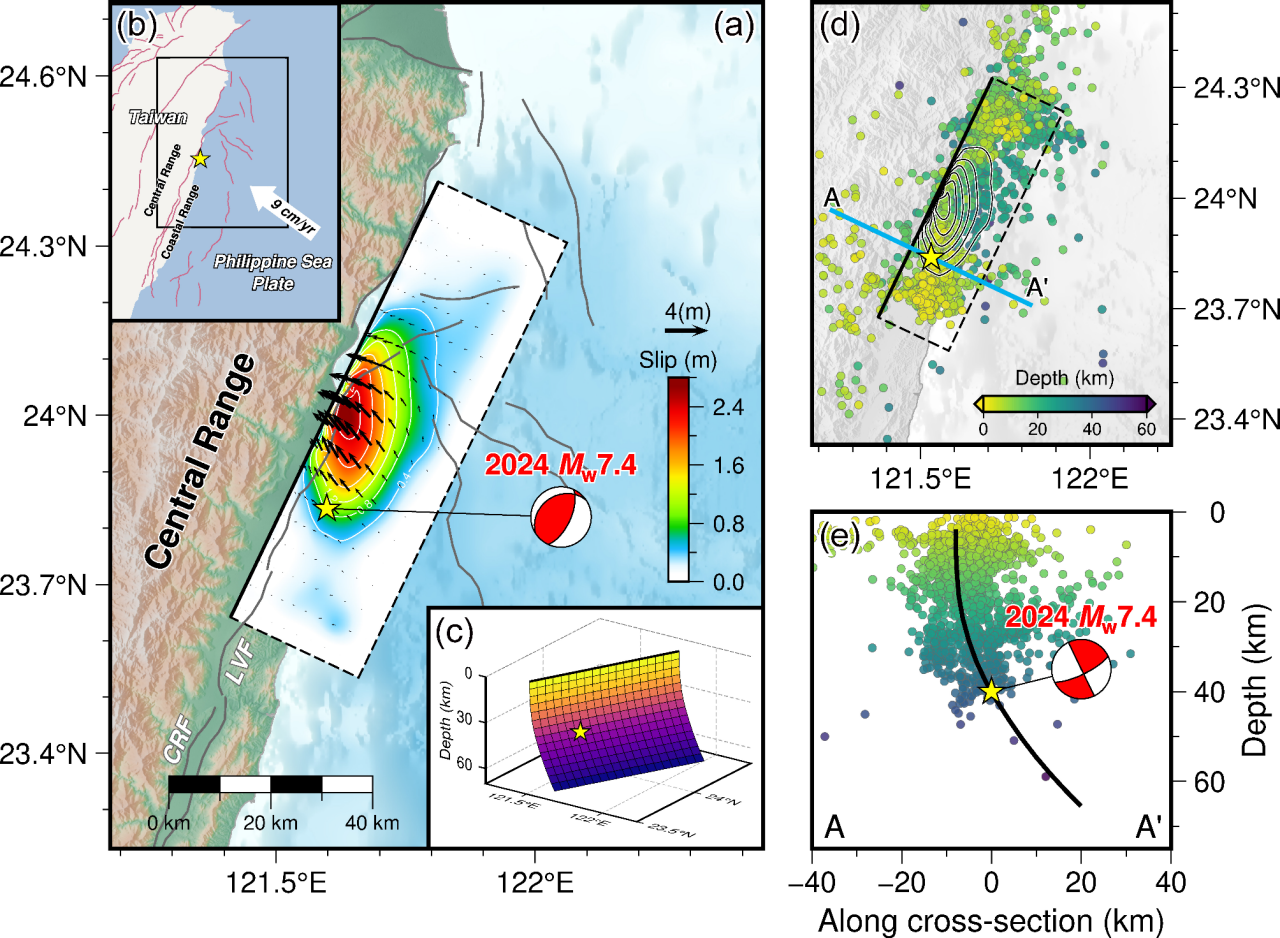
(**a**) Map showing the study area for the 2024 Hualien earthquake. The yellow star indicates the epicenter. The active faults retrieved from Zelenin et al.^56^ are shown in gray lines, including the Longitudinal Valley fault (LVF) and the Central Range fault (CRF). The slip distribution and the ground projection of our preferred finite-fault model for the Hualien earthquake are shown within the black frame in the map view, with the solid line indicating the top edge. The cumulative moment tensor derived from the joint finite-fault inversion is plotted as the beach ball in red. (**b**) Map showing the position of the study area for the Hualien earthquake, which is indicated by the black frame. The white arrow represents the convergence direction and rate between the Eurasian and Philippine Sea plates. (**c**) Our preferred fault model with a listric geometry used for reconstructing the rupture process of the Hualien earthquake. The colors represent the fault dip that decreases gradually from 90° to 45° with the increasing depth. The yellow star indicates the hypocenter. (**d**) Aftershocks following the mainshock in one month, which are collected from the Taiwan Seismological and Geophysical Data Management System (GDMS), are plotted in circles with the colors corresponding to the focal depths. The blue line is the cross-section A-A’ that is perpendicular to the strike of the seismogenic fault. The black lines represent the contours of the slip distribution. (**e**) Cross-section A-A’ with the aftershocks projected into it. The black line indicates the fault plane projected into the cross-section. The cumulative moment tensor is also plotted in the side view. The figures were generated by the Generic Mapping Tools 6.3.0 (GMT 6.3.0, https://www.generic-mapping-tools.org).

Over 2,300 aftershocks of magnitude larger than 1.0 were reported by the Central Weather Administration (CWA) within one month after the mainshock, which are concentrated along the eastern edge of the Longitudinal Valley (LV) suture above the depth of 50 km (Fig. 1). The distribution of the 2024 Hualien sequence is similar to the NE-trending LV, and is consistent with the possible fault orientation in the USGS-NEIC, Global Centroid Moment Tensor (GCMT), and Broadband Array in Taiwan for Seismology (BATS) solutions (Table S1). The elongated LV was formed from the collision of the Eurasian and Philippine Sea plates, where the east-dipping Longitudinal Valley fault (LVF) and the west-dipping Central Range fault (CRF) have developed^1–3^. As the LV accommodates about one third of the convergence rate of approximately 9 cm/yr, it becomes an active suture zone and also one of the most seismically active regions in Taiwan^4,5^. Despite several major earthquakes have occurred along the LV in the recent decade^6–15^, the 2024 Hualien earthquake has become the largest one in this region since the 1951 Longitudinal Valley earthquake sequence, which included four earthquakes with magnitude greater than 7.0 that occurred along the east-dipping LVF^16,17^. The occurrence of the 2024 event naturally raises attentions and worries about the possibility of whether other large earthquakes would continue to occur like the 1951 sequence. As the largest earthquake in Taiwan since the 1999 Chi-Chi *M*_w_ 7.6 earthquake^18,19^, it is necessary to take advantage of the unique opportunity provided by the 2024 Hualien earthquake to illuminate the rupture behavior of current large earthquakes in the northeastern LV, and to further investigate the potential triggering mechanism for the future seismic hazard.

Therefore, in this study, we collected all available geodetic and seismic observations to reconstruct the rupture kinematics of the 2024 Hualien earthquake. We also examined the influences imposed by historical major earthquakes on the 2024 event, and the regional seismic hazard in the future was discussed based on the induced Coulomb failure stress (CFS) changes.

## Results

### Listric fault geometry

To retrieve the possible source parameters for the 2024 Hualien earthquake, we conducted a Bayesian estimation using geodetic data with loose constraints. Nevertheless, the nonlinear inversion under the prior assumption of uniform slip cannot reflect the inherent heterogenous feature of the source rupture due to the inevitable trade-offs between fault width, depth, and dip slip (Fig. S3). Hence the further inversion of slip distribution or even the rupture process is imperative. As an effective indicator of the fault geometry or orientation for the mainshock^20^, the aftershocks of the Hualien earthquake trends from SSW to NNE in the map view (Fig. 1), and are closer to a steeply SE-dipping fault, which is consistent with the optimal source parameters obtained by the Bayesian estimation, but all the released focal mechanism solutions indicate a fault dip not exceeding 60° (Table S1). Therefore, we further searched for the optimal dip within the range of 10°–90° at an interval of 1° through the finite-fault inversion using the geodetic observations, including the static global navigation satellite system (GNSS) displacements and interferometric synthetic aperture radar (InSAR) data. Since the fault dimension of the uniform slip model is probably difficult to accommodate the heterogenous fault slip, we fixed the fault orientation at the optimal strike of 25.4° obtained by the Bayesian estimation, and enlarged the rectangular fault to 95 km × 70 km to satisfy the finite-fault inversion. The grid search indicates an optimal dip of 72°–73° (Fig. S10), which is consistent with the Bayesian estimation. Subsequently, we conducted a set of finite-fault inversions of teleseismic *P* waves, separately using the optimal dip of 72.8° derived from the Bayesian estimation and the dip of 54.0° indicated by the SE-dipping nodal plane in the USGS-NEIC moment tensor solution. However, the fit to the waveforms in a specific azimuthal range for the inversion using the high dip angle are not as good as another inversion (Fig. S11 and S12), which blurs the determination of fault dip. Different types of observed data have diverse imaging capabilities for source characteristics. Geodetic observations, such as the surface deformation measured by GNSS and InSAR, significantly attenuate with an increasing epicentral distance, making the near-field observations more sensitive to the fault geometry or slip details at shallow depths^21^. In contrast, the teleseismic *P* waves retain the low-frequency component through the far-field attenuation, reflecting the overall characteristics of the source rupture^22,23^. Therefore, the discrepancy between the results of the aforementioned finite-fault inversions using various types of data implies the complexity of the fault geometry associated with the Hualien earthquake, represented by the varying dip angle with depth. Given the relative consistency between the fault strike delineated by the aftershock distribution, Bayesian estimation and moment tensor solutions, the Hualien earthquake is likely to align with the strike of the east-dipping LVF and occur on the northward extension of this fault. Based on the listric geometry with varying dip of the LVF developed by the Taiwan Earthquake Model (TEM)^24^, we set the fault dip to decrease gradually from 90° to 45° with the increasing depth, while fixing the strike as that obtained by the Bayesian estimation, also ensuring the hypocenter located by the USGS-NEIC within the fault plane. Such a listric fault is probably a composite formed by the subduction between the Luzon volcanic arc lithosphere and the forearc sliver, which includes both the shallow reverse fault wedge and the contact interface between the subducting lithospheres^25^. The fault geometry also matches well with the aftershock distribution (Fig. 1), in which a series of subfaults with a size of 5 km × 5 km were divided for the fault parameterization.

### Temporospatial rupture kinematics

**Fig. 2:**
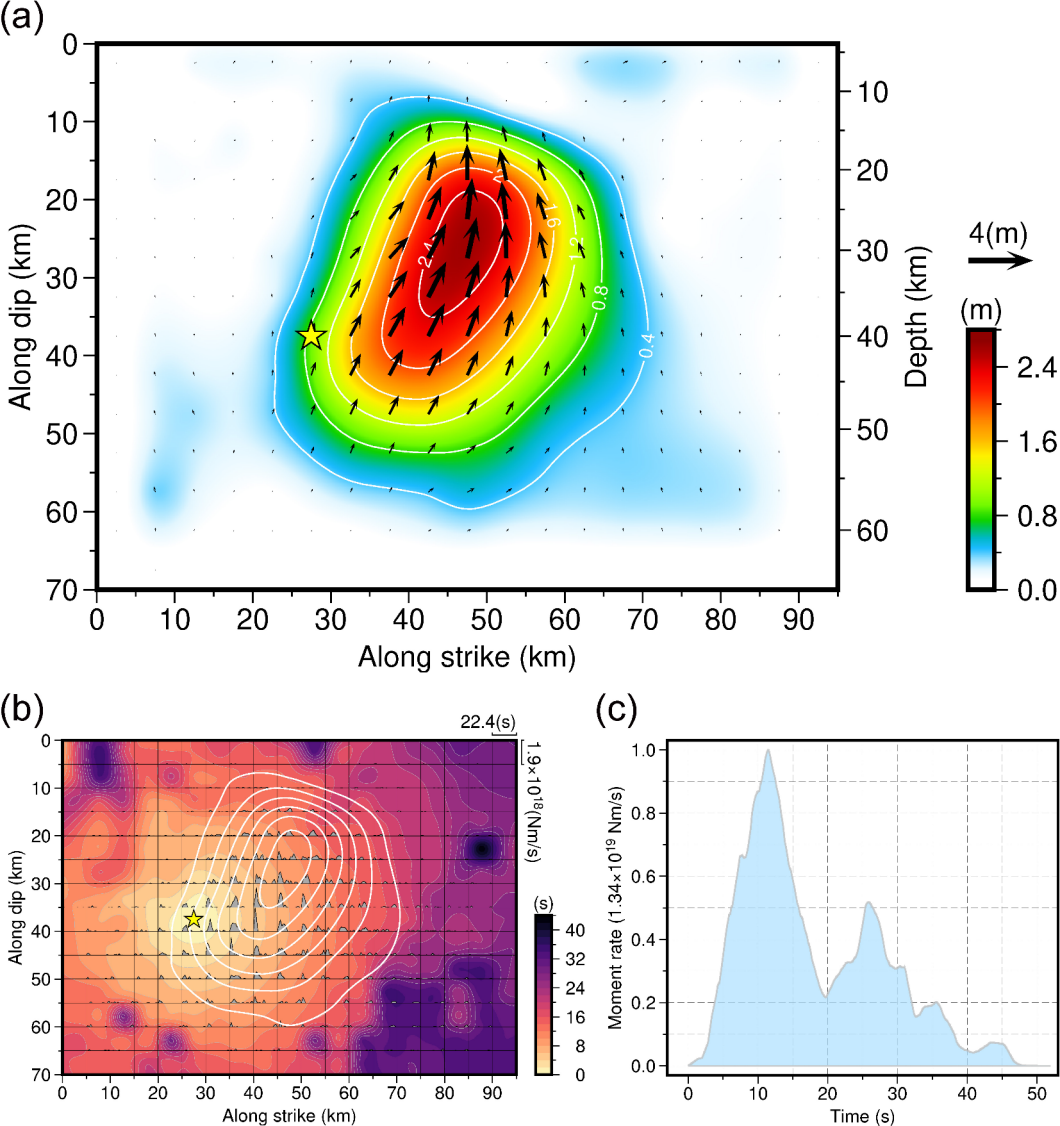
Results of the joint finite-fault inversion for the 2024 Hualien earthquake. (**a**) Slip distribution of the Hualien earthquake, with the slip contours at an interval of 0.4 m. The yellow star indicates the hypocenter. (**b**) Seismic moment releasing rate functions of the subfaults on the fault plane, with the rupture front propagation shown as the background color and the slip contours plotted as white lines. (**c**) Total seismic moment releasing rate function of the Hualien earthquake. The figures were generated by the Generic Mapping Tools 6.3.0 (GMT 6.3.0, https://www.generic-mapping-tools.org).

**Fig. 3:**
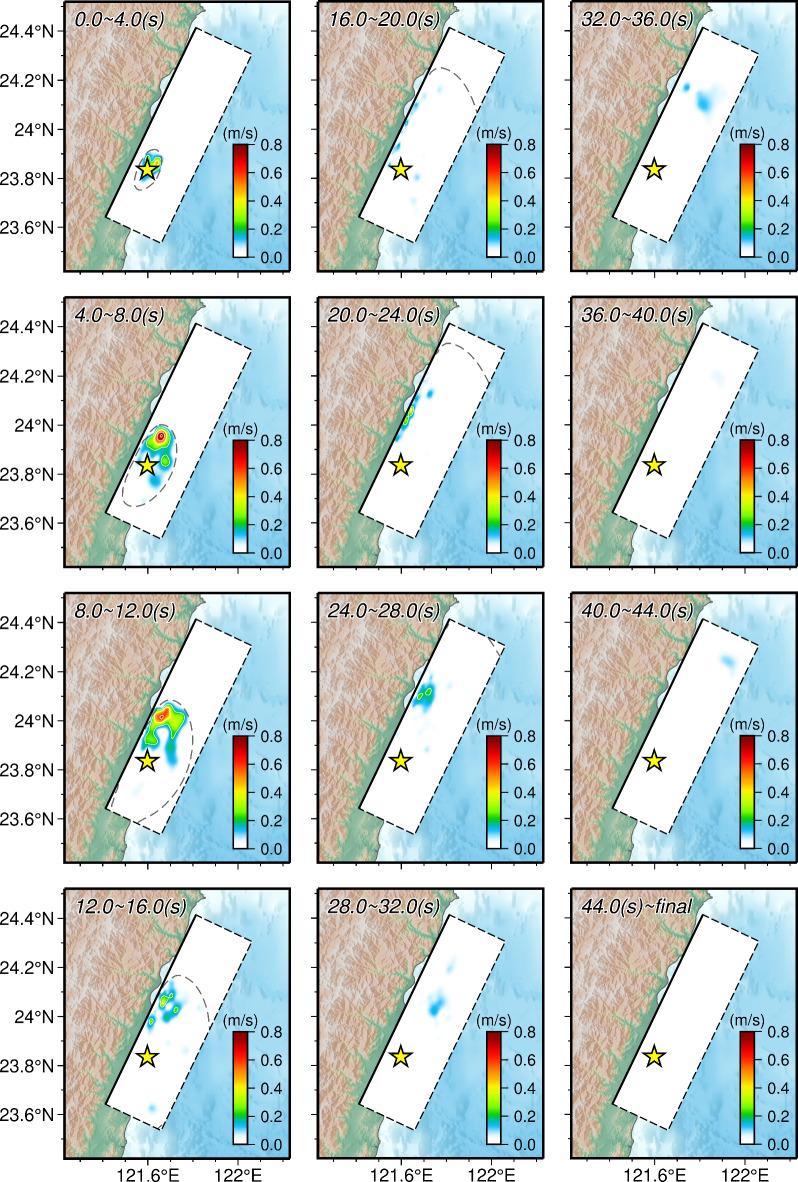
Source rupture process represented by the slip rate evolution of our preferred fault model in the map view. The contour interval of slip rate is 0.2 m/s. The yellow star indicates the epicenter. The rupture front defined by a rupture velocity of 2.5 km/s is represented by the gray dashed line. The figures were generated by the Generic Mapping Tools 6.3.0 (GMT 6.3.0, https://www.generic-mapping-tools.org).

We constructed the kinematic rupture process of the 2024 Hualien earthquake through a joint finite-fault inversion combining geodetic and seismic observations. Our preferred finite-fault model reveals that the Hualien earthquake occurred on a SE-dipping fault with a listric geometry (Fig. 1). Since no significant deformation discontinuities attributed to surface ruptures were observed in the InSAR images, this listric fault was set to not reach the ground surface. The slip distribution of the Hualien earthquake exhibits a compact rupture pattern dominated by thrust motions (Fig. 2), with only one asperity situated in the middle of the fault and on the NE of the hypocenter. The asperity extends about 45 km along the fault strike and about 50 km along the down-dip direction, with a peak slip of up to 2.58 m and an average slip of about 1.15 m, which is similar to the uniform slip model of the Bayesian estimation. However, the slips in the uppermost area of the fault are not obvious, suggesting that the Hualien earthquake is a unilateral rupture primarily extending to the NNE on a blind fault, also aligning with the backprojection imaging analysis (Fig. S8 and S9). Additionally, the existence of aftershocks both internally and surrounding the main slip zone suggests that the rupture pattern of the mainshock may be related to the occurrence of aftershocks (Fig. 1). As the depth increases, the distribution extent of aftershocks along the fault strike is gradually shrinking (Fig. S15). The aftershocks at depths are mainly located within the asperity area, suggesting the probable existence of afterslip in the main slip zone. In contrast, the aftershocks in the shallow portion of the fault are located outside the asperity and are relatively more concentrated, indicating that they are more likely to be triggered by the mainshock. It requires further in-depth investigation to clarify whether the triggering effect is achieved through the CFS change or the migration of fluid content induced by the mainshock. The rupture process of the Hualien earthquake shows that the release of seismic energy can be divided into three stages (Figs. 2 and 3). From 0.0 to 19.0 s, the rupture is initiated at the hypocenter, and its propagation front expands in a nearly circular pattern, with the rupture mainly extending laterally to the NNE. This stage is the one that releases the most seismic moment throughout the entire rupture, reaching a peak slip rate of 0.84 m/s at about 6.0 s and a maximum seismic moment releasing rate of 1.34 × 10^19^ Nm/s at about 12.0 s. From 19.0 to 33.0 s, the rupture continues to expand toward the NNE and also propagates along the up-dip direction, but there appears to be a delay in the rupture that is later than the fastest propagation front. During this stage, the moment releasing rate function shows a second peak at about 26.0 s. After 33.0 s, the fastest rupture front has crossed the entire fault plane, but there is still ongoing rupture in the NE along the down-dip direction. The duration of the entire source rupture is approximately 48.0 s, releasing a scalar seismic moment of 2.09 × 10^20^ Nm (*M*_w_ 7.48), which is similar to the uniform slip model of the Bayesian estimation. The cumulative seismic moment tensor indicates a hybrid focal mechanism of thrust and sinistral strike-slip faulting with strike/dip/rake = 25.0°/64.0°/75.0°, which is close to the moment tensor solutions determined by the USGS-NEIC and GCMT. To estimate the uncertainty of the finite-fault model, we conducted a Jackknife test through the repeated inversion of 100 trials, in which 20% of the observed data were randomly removed in each trial. The Jackknife test indicates that the maximum uncertainty of fault slip is 0.25 m at the 95% confidence level (Fig. S16), demonstrating that the features of the slip distribution derived from the joint finite-fault inversion are persistent.

## Discussion

### Triggering effect of historical events

**Fig. 4:**
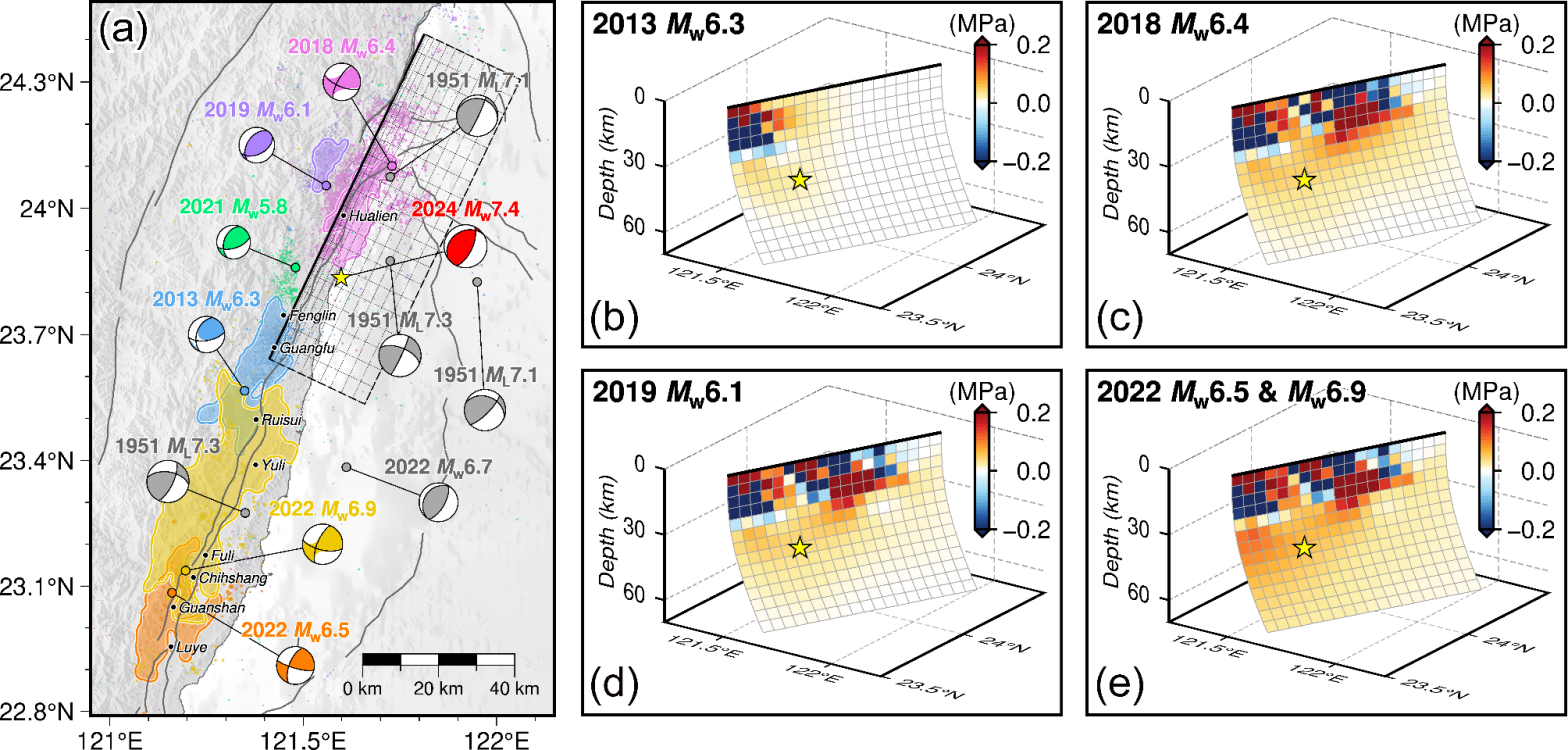
(**a**) Map showing the historical major events along the Longitudinal Valley (LV). The colored areas are the main slip zones of the historical major events^6–9^. The epicenter locations of the historical events are collected from the GDMS, and the corresponding moment tensor solutions are determined by the U.S. Geological Survey-National Earthquake Information Center (USGS-NEIC). For the four events in the 1951 Longitudinal Valley earthquake sequence, the epicenters and moment tensor solutions are collected from Chen et al.^16^. The cumulative moment tensor of the 2024 Hualien earthquake is plotted as the beach ball in red. (**b**–**e**) Cumulative Coulomb failure stress (CFS) changes induced by the representative historical major events on the seismogenic fault of the 2024 Hualien earthquake after (**b**) the 2013 Ruisui *M*_w_ 6.3 earthquake, (**c**) the 2018 Hualien *M*_w_ 6.4 earthquake, (**d**) the 2019 Hualien *M*_w_ 6.3 earthquake, and (**e**) the 2022 Chihshang *M*_w_ 6.5 and *M*_w_ 6.9 earthquakes. The figures were generated by the Generic Mapping Tools 6.3.0 (GMT 6.3.0, https://www.generic-mapping-tools.org).

Over the past decade, there have been several major earthquakes with plenty of aftershocks along the LV. The occurrence of an earthquake will affect the adjacent seismicity, and the CFS change can quantify this effect. The positive CFS change represents the stress loading that promotes the occurrence of subsequent earthquakes, and vice versa. To elucidate the possible influence of the historical events on the occurrence of the 2024 Hualien earthquake, we analyzed the static CFS changes caused by several representative major earthquakes, including: the 2013 Ruisui *M*_w_ 6.3 earthquake^6,10^, the 2018 Hualien *M*_w_ 6.4 earthquake^7,11–13^, the 2019 Hualien *M*_w_ 6.3 earthquake^8,14^, and the 2022 Chihshang *M*_w_ 6.5 and *M*_w_ 6.9 earthquakes^9,15^. Utilizing the Coulomb 3.3 software^26^, the cumulative CFS changes of our preferred fault model were calculated based on the slip models of historical events^6–9^, with an effective friction coefficient of 0.4 describing the effect of pore fluids and the material properties of the fault zone^27^. As shown in Fig. 4, since the major slips of these historical events are all above the depth of 30 km, the corresponding significant CFS changes also concentrate on the shallow portion of the seismogenic fault of the 2024 Hualien earthquake, with the primary contributions coming from the 2013 Ruisui earthquake and the 2018 Hualien earthquake. The CFS changes imposed by the 2013 Ruisui earthquake are confined to the southern shallow part of the causative fault, while the influence of the 2018 Hualien earthquake is located in the middle section. The features show a close relation to the slip patterns of the two earthquakes. In addition, these historical earthquakes all produce substantial stress loading on the remaining area of the seismogenic fault, except that the 2019 Hualien earthquake generates negative CFS changes that are nearly negligible. Specifically, the 2018 Hualien earthquake produces a CFS increment of about 0.023 MPa at the hypocenter of the 2024 event, exceeding the classical threshold for earthquake triggering of 0.01 MPa^28^. While the two Chihshang earthquakes in 2022, which are the most distant from the 2024 Hualien earthquake, also increase the CFS at the hypocenter by a similar amount of 0.022 MPa, but produce a more intense stress loading in the adjacent area. Considering that the Chihshang earthquakes are the most recent of the listed historical events, we suggest that they have the most significant triggering effect on the 2024 Hualien earthquake. Secondly, the impact imposed by the 2018 Hualien earthquake should not be neglected.

### Regional seismic hazard evaluation

**Fig. 5:**
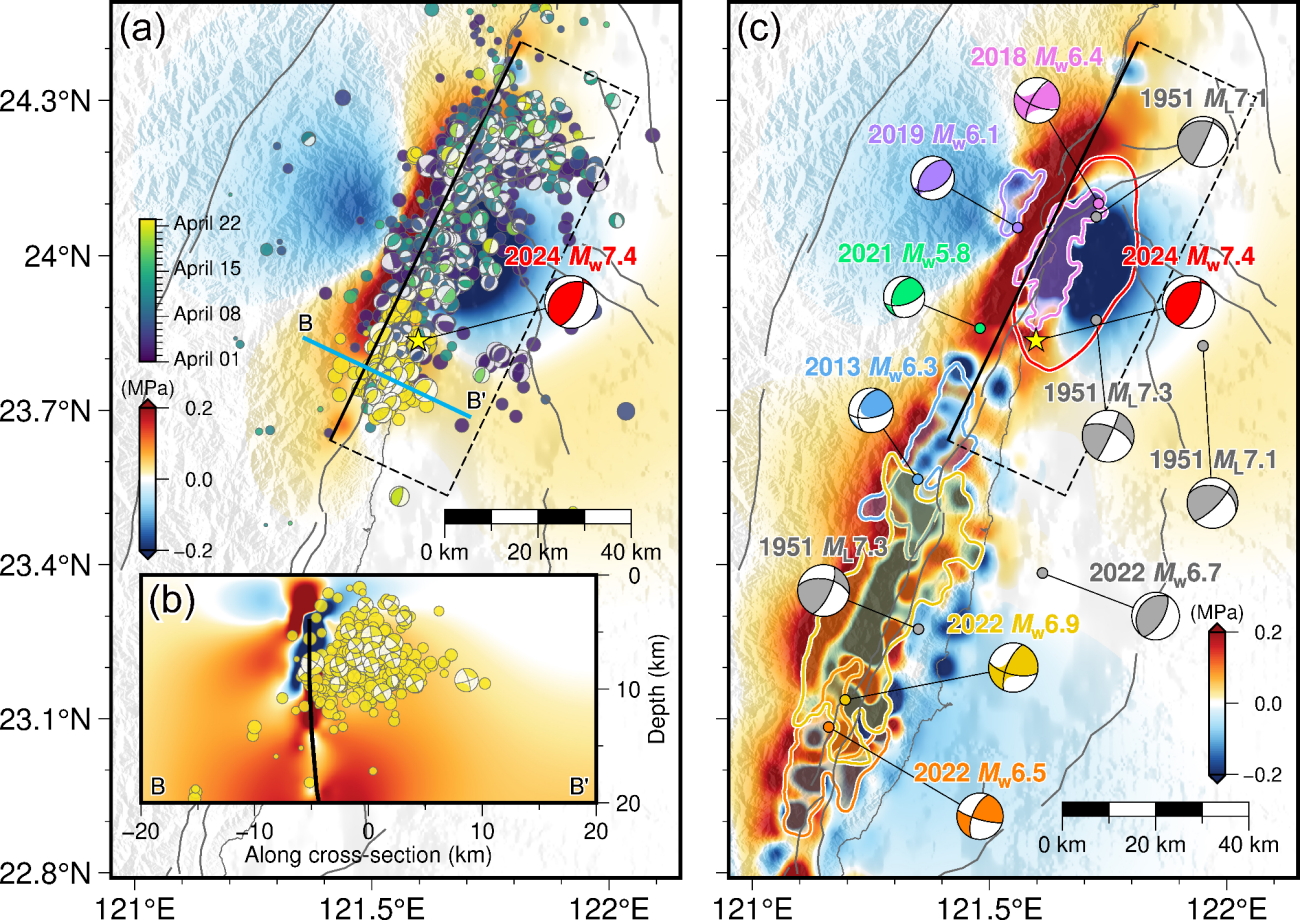
(**a**) Regional field of the CFS changes generated by the 2024 Hualien earthquake. The aftershocks following the mainshock until 22 April 2024 are plotted in circles, and the moment tensors of some aftershocks collected from the Broadband Array in Taiwan for Seismology (BATS) solutions are plotted in beach balls, with the colors corresponding to the focal depths. The blue line is the cross-section B-B’ that is perpendicular to the strike of the seismogenic fault. (**b**) CFS changes in the cross-section B-B’ generated by the 2024 Hualien earthquake. The aftershocks of the seismic swarm on 22 April 2024 with a few moment tensor solutions are projected into the cross-section. The black line indicates the fault plane projected into the cross-section. (**c**) Regional field of the cumulative CFS changes induced by the representative historical major earthquakes since 2013, including the 2024 Hualien earthquake. The figures were generated by the Generic Mapping Tools 6.3.0 (GMT 6.3.0, https://www.generic-mapping-tools.org).

Numerous aftershocks followed the *M*_w_ 7.4 mainshock of the 2024 Hualien sequence. The aftershock activity usually weakens over time, but notably, it reached another active peak on 22 April 2024 (Fig. S21) at the southern termination of the mainshock rupture. The events are concentrated above the depth of 20 km, and are mainly dominated by the thrust faulting, which are similar to the focal mechanism of the mainshock. These aftershocks that occurred continuously in such a short period are likely to originate from a similar source and formed a seismic swarm. To explore the potential correlation between the seismic swarm and the mainshock, we analyzed the regional field of the CFS changes induced by the mainshock, which is the average within the depth of 0 to 20 km, calculated from our preferred finite-fault model with an effective friction coefficient of 0.4, corresponding to a thrust receiver fault with the orientation (strike/dip/rake = 25.0°/64.0°/90.0°) consistent with the cumulative moment tensor of the mainshock. As shown in Fig. 5, the majority of aftershocks exists in the area of increased CFS or the transition zone between stress loading and unloading, reflecting the modulation on the emergence of aftershocks due to the rupture pattern of the mainshock. In contrast, the seismic swarm is almost entirely in the area of positive CFS changes, which is also demonstrated in the cross-section perpendicular to the fault strike of the mainshock. This indicates that the seismic swarm is likely associated with the stress loading caused by the mainshock, but its occurrence about 20 days after the mainshock further implies that the increased CFS may not directly exert a triggering effect. The appearance of a seismic swarm, compared to typical aftershocks, is attributed not only to stress triggering but also controlled by other factors^29^. For instance, the fluid pressure perturbations resulted from the upwelling of crustal fluids drove the spatiotemporal migration of a seismic swarm that persisted for as long as five years beneath the Noto Peninsula in Japan^30–32^. The upward fluids dehydrated from the subducting slab has been verified to exist within the crust along the LV, and its invasion may have reached the northward extension of the LVF, and further triggering the 2018 and 2019 Hualien earthquakes^33,34^. For the nonplanar fault in the 2024 Hualien earthquake, the void space in the vicinity caused by the rupture could significantly increase the permeability, thereby enabling the upward migration of fluid pressure^35,36^. Therefore, such fluid pressure perturbations under the stress loading produced by the mainshock may lead to the intensive occurrence of regional seismic activities, thus forming the seismic swarm on 22 April.

Furthermore, the regional field of the cumulative CFS changes generated by the representative major earthquakes since 2013, including the 2024 Hualien earthquake, was also analyzed and illustrated in Fig. 5. For the 2024 event, the influence exerted on the CFS changes of the slip uncertainties indicated by the Jackknife test was also taken into account (Fig. S22). The significant stress loading is mainly concentrated in the northern LV, surrounding the main rupture area of the 2024 event. In the ruptures of historical events such as the 2018 Hualien earthquake and the two Chihshang earthquakes in 2022, the involvement of both the east-dipping LVF and the west-dipping CRF were contained. The causative fault of the 2024 Hualien earthquake is clearly more related to the LVF, and previous analyses reflect that the release of seismic energy is concentrated on the deeper part. While the CRF has been ruptured in the 2018 and 2019 Hualien earthquakes^7,8,13,14^, it is no longer capable of hosting or being triggered for shallow slips, thus currently making it difficult to increase the corresponding seismic hazard. The main slip areas of recent major earthquakes along the LV are located on the west-dipping CRF, while the east-dipping LVF has experienced a relative quiescence of major earthquakes since the 1951 Longitudinal Valley earthquake sequence, until the 2018 Hualien earthquake and the 2022 Chihshang earthquakes. According to the seismic moment budget estimated by Tang et al.^15^ based on the centennial earthquake records and millennial geologic rates, the surplus on the LVF before the 2024 event is 1.7–3.1 × 10^20^ Nm, equivalent to an earthquake of *M*_w_ 7.4–7.6. Hence the 2024 Hualien earthquake may not completely release the accumulated seismic moment, even though sporadic *M*_w_ ≥ 6.0 earthquakes have taken place along the deep part of the LVF. In particular, there has been a long-term absence of major earthquakes in the area between the ruptures of the 2013 Ruisui earthquake and the 2018 and 2024 Hualien earthquakes. The interseismic coupling model of the LVF^37^ also reveals that the main locking is concentrated on its northernmost section, where only minor slips occurred in the 2024 earthquake, so the insufficiently ruptured area has the potential for hosting major earthquakes in the future. Furthermore, the overall faulting mechanism of the 1951 sequence transformed from being dominated by left-lateral strike-slip with a thrust component into thrusting dominantly from NNE to SSW^17^. While the focal mechanism of the 2024 Hualien earthquake is opposite to that of the northern rupture area of the 1951 sequence, implying that the release of the accumulated strain on the LVF due to the oblique arc-continent collision is manifested as the complementary dominance of strike-slip or thrust faulting in multiple earthquake episodes, and may be influenced by the differences in the focal depths. Therefore, the potential earthquakes are also likely to follow this controlling pattern of focal mechanism.

### Summary

The 2024 Hualien earthquake occurred on a SE-dipping blind listric fault associated with the northward extension of the LVF, manifesting as a unilateral rupture that mainly extends toward the NNE. The slip distribution shows a compact rupture pattern, primarily characterized by the thrust faulting, with a peak slip of approximately 2.58 m. The source rupture process lasts for about 48.0 s and releases a scalar seismic moment of 2.09 × 10^20^ Nm (*M*_w_ 7.48). The analysis of the induced CFS changes suggests that the two Chihshang earthquakes in 2022 produce evident stress loading in the nucleation zone of the 2024 Hualien earthquake, which has a triggering effect on the 2024 event. The coupled effects of the increased CFS and the possible upward invasion of crustal fluids attributed to the 2024 Hualien earthquake, leads to a seismic swarm that occurred 20 days after the mainshock at the southernmost tip of the rupture area. Furthermore, considering that the accumulated seismic moment on the LVF may still not be fully released after the 2024 earthquake, the seismic hazard in the insufficiently ruptured area between the main ruptures of the 2013 Ruisui earthquake and the 2024 Hualien earthquake deserves further attention in the future.

## Methods

### Geodetic data processing

We used the synthetic aperture radar (SAR) data acquired by the L-band LuTan-1 (LT-1) satellite from the Natural Resources Satellite Remote Sensing Cloud Service Platform, and the C-band Sentinel-1 satellite operated by the European Space Agency (ESA). The LT-1 data were acquired from the ascending orbit in the stripmap mode. The Sentinel-1 data were acquired from the ascending track 69 and the descending track 105 in the terrain observation by the progressive scans (TOPS) mode (Table S2). All SAR data were processed using the GAMMA software^38^. To obtain the line-of-sight (LOS) deformation field of the 2024 Hualien earthquake, we performed differential interferometric synthetic aperture radar (DInSAR) processing on the LT-1 and Sentinel-1 data (Fig. S1). To improve the signal-to-noise ratio (SNR), we set 5 range and 5 azimuth looks for the LT-1 data, and 20 range and 5 azimuth looks for the Sentinel-1 data. The Shuttle Radar Topographic Mission (SRTM) digital elevation model (DEM) of 30 m resolution was used to coregister single look complex (SLC) image pairs and eliminate the topographic phase in the interferograms^39^. After filtering the interferograms with the Goldstein filter method^40^, the minimum cost flow method^41^ was used to unwrap phase by masking the areas with discontinuous interference fringes and coherence value lower than 0.3. The near-field unwrapping errors were manually corrected. The long-wavelength atmospheric and orbital errors were removed through a polynomial fitting, which was estimated from the stable far-field observations. Finally, we subsampled the unwrapped LOS displacements using the quadtree method^42^. The static three-component displacements of GNSS sites for the 2024 earthquake from 5-minute sample rate time series derived with rapid orbits by the Jet Propulsion Laboratory (JPL) were also included in the analysis (Fig. S7 and S18).

### Seismic data processing

The seismic dataset in this study utilized the strong-motion waveforms and the teleseismic body-wave records. The strong-motion records contain three-component acceleration waveforms of 26 stations within 100 km of the epicenter (Fig. S2), which were acquired from the Taiwan Strong Motion Instrument Program (TSMIP). We preprocessed the initial strong-motion records, including band-pass filtering between 0.1 and 0.4 Hz and resampling at 5 Hz, followed by integrating once to convert to velocity waveforms. Since the 2024 Hualien earthquake occurred in the offshore area of Taiwan, the used onshore strong-motion stations are almost located on the west of the epicenter. To increase azimuthal coverage and diversify the data types, 40 teleseismic *P*-wave records with epicentral distances ranging from 30° to 90° were also included in the study (Fig. S2), which were collected from the Data Management Center of the Incorporated Research Institutions for Seismology (IRIS DMC). The displacement waveforms were produced by removing the instrumental response from the initial teleseismic records and were subsequently band-pass filtered between 0.005 and 0.5 Hz, as well as resampled at 5 Hz.

### Joint finite-fault inversion

The temporospatial rupture kinematics of the 2024 Hualien earthquake was derived from the joint finite-fault inversion utilizing an integrated dataset, including the InSAR images, static GNSS displacements, teleseismic *P* waves, and three-component strong-motion records. A horizontally layered structure for the source region spanning from near-field to regional scales was extracted from Huang et al.^43^, which was used to generate the Green’s functions of regional observations, including the InSAR, GNSS and strong-motion data via the FK code^44^. The Multitel3 code^45^ was employed to generate the Green’s functions of teleseismic *P* waves depending on the CRUST1.0 model^46^. Following the finite-fault inversion strategy of the linear multi-time-window method^47,48^, a linear equation connecting the discretized source parameters with the observed data can be established via the representation theorem of Aki and Richards^49^ as follows,

1 $$\:\left[\begin{array}{c}\mathbf{G}\\\:\lambda\:\mathbf{S}\end{array}\right]\mathbf{m}=\left[\begin{array}{c}\mathbf{d}\\\:\mathbf{0}\end{array}\right]$$

Here, $\:\mathbf{G}$ is the Green’s function matrix convolved with the time-window function, $\:\mathbf{m}$ is the unknown vector of fault slips, and $\:\mathbf{d}$ is the vector of observed data used in the inversion. To ensure the stability of the linear inversion, a modified form of Laplacian smoothing constraint^50^ represented by matrix $\:\mathbf{S}$ was introduced, and $\:\lambda\:$ is the factor describing the smoothing strength. Based on the positivity constraint, the linear equation is commonly solved through the nonnegative least-squares algorithm^51^, in which the slip direction is limited within ± 45° of a main rupture direction. Nevertheless, in this study, we introduced the inequality constraint of Xu^52^ to relax and enable the fault slip to vary within the half-space confined by the main rupture direction, which can accommodate more complicated variations of source mechanism. The inequality constraint is described as,

2 $$\:{\mathbf{n}}_{k}^{T}{\mathbf{m}}_{k}\ge\:0,\:\left(k=\text{1,2},\cdots\:,K\right)$$

where $\:{\mathbf{n}}_{k}$ represents the unit vector of the main rupture direction prescribed for the $\:k$-th subfault, and $\:{\mathbf{m}}_{k}$ is the slip vector of the corresponding subfault. The inequality constraint is equivalent to a matrix form as $\:\mathbf{B}\mathbf{m}\ge\:0$, where $\:\mathbf{m}$ is the unknown vector of all subfaults on the fault plane, and the diagonal matrix $\:\mathbf{B}$ is shown as follows,

3 $$\:{\mathbf{B}} = \left[ {\begin{array}{*{20}c} {{\mathbf{n}}_{1}^{T} } & {\:0} & {\: \cdots \:} & {\:0} \\ {\:0} & {\:{\mathbf{n}}_{2}^{T} } & {\: \cdots \:} & 0 \\ {\: \vdots } & {\: \vdots } & {\: \ddots \:} & {\: \vdots } \\ {\:0} & {\:0} & {\: \cdots \:} & {\:{\mathbf{n}}_{K}^{T} } \\ \end{array} } \right]$$

Thus, the regularized inverse problem with the inequality constraint to be solved is,

4 $$\:{\text{min:}}\left( {{\mathbf{d}} - {\mathbf{Gm}}} \right)^{T} \left( {{\mathbf{d}} - {\mathbf{Gm}}} \right) + \lambda \:^{2} {\mathbf{m}}^{T} {\mathbf{Sm}}$$

Without imposing the inequality constraint, the least-squares algorithm can be used to obtain,

5 $$\:{\widehat{\mathbf{m}}}_{0}={\mathbf{N}}^{-1}{\mathbf{G}}^{T}\mathbf{d}$$

where $\:\mathbf{N}={\mathbf{G}}^{T}\mathbf{G}+{\lambda\:}^{2}{\mathbf{S}}^{T}\mathbf{S}$. Following Van de Panne^53^, the solution with the applied inequality constraint can be directly written as,

6 $$\:\widehat{\mathbf{m}}={\widehat{\mathbf{m}}}_{0}+{\mathbf{N}}^{-1}{\mathbf{B}}^{T}\mathbf{q}$$

The vector $\:\mathbf{q}$ can be solved through a linear complementarity problem^54^,

7 $$\:\mathbf{h}=\mathbf{B}{\mathbf{N}}^{-1}{\mathbf{B}}^{T}\mathbf{q}+\mathbf{B}{\widehat{\mathbf{m}}}_{0}$$

where $\:{\mathbf{h}}^{T}\mathbf{q}=0$, $\:\mathbf{h}\ge\:0$, and $\:\mathbf{q}\ge\:0$. The rupture velocity and the rise time of time-window were tested through a series of inversion trials due to their inescapable trade-off (Fig. S13). By examining the fit to the observed data, the rupture velocity was selected as 2.5 km/s from the range from 1.5 to 3.5 km/s with an interval of 0.1 km/s, which also matches well with the backprojection results of multiple arrays (Fig. S9), and the rise time of 2.8 s was selected from the range from 0.8 to 6.0 s with an interval of 0.4 s. Each subfault contains 15 half-overlapping triangle time-windows to account for variations in the slip history and rupture velocity, and the slip rake is limited within 74° ± 90° according to the USGS-NEIC moment tensor solution. The Frobenius norm was initially used to normalize all the datasets^55^, and then the InSAR images, static GNSS displacements, teleseismic *P* waves, and strong-motion waveforms were separately weighted by the preferred scheme of 1.00, 0.20, 0.15, and 0.15. The smoothing factor was selected as 0.0012 according to the classic method of L-shaped curve (Fig. S13).

## Electronic supplementary material

Below is the link to the electronic supplementary material.

Supplementary Material 1

## Data Availability

The teleseismic waveforms were collected from https://ds.iris.edu/wilber3/find_stations/11824102. The TSMIP strong-motion data were downloaded from the Taiwan Seismological and Geophysical Data Management System (GDMS, https://gdms.cwa.gov.tw/event.php?id=37). The Sentinel-1 data were downloaded from the Sentinel-1 Scientific Data Hub (https://scihub.copernicus.eu). The LT-1 data were provided by the China Centre for Resources Satellite Data and Application. The static GNSS offsets were provided by the Nevada Geodetic Laboratory (NGL, http://geodesy.unr.edu). The aftershock catalog of the 2024 Hualien earthquake was retrieved from GDMS (https://gdms.cwa.gov.tw/catalogDownload.php), and the BATS solutions of aftershocks were collected from https://tecdc.earth.sinica.edu.tw/FM/AutoBATS. The unwrapped InSAR images and the finite-fault slip model associated with the 2024 Hualien earthquake in this study can be downloaded from 10.5281/zenodo.12724251.
