# Supplementary material for: Thrust-dominated unilateral rupture of a blind listric fault associated with the 2024 Hualien earthquake
Source: Sci Rep. 2024 Dec 28;14:31496. doi: 10.1038/s41598-024-82971-x (PMC11682310; doi:10.1038/s41598-024-82971-x)
Supplement: Supplementary file 1 — Supplementary Material 1 [file 41598_2024_82971_MOESM1_ESM.pdf]

Supporting Information for

Thrust-dominated unilateral rupture of a blind listric fault associated with the 2024 Hualien earthquake

Ao Zheng[1,2*], Xiangwei Yu[1], Chengyuan Bai[3], Wenbin Xu[3], Jiaqi Qian[1], Wenbo Zhang[1], Xiaofei Chen[4]

[1]College of Earth and Planetary Sciences, University of Chinese Academy of Sciences, Beijing, China

[2]School of Resources and Geosciences, China University of Mining and Technology, Xuzhou, China

[3]School of Geosciences and Info-Physics, Central South University, Changsha, China

[4]Department of Earth and Space Sciences, Southern University of Science and Technology, Shenzhen, China

Contents of this file

Supplementary Notes S1 to S2
Supplementary Figures S1 to S22
Supplementary Tables S1 to S3

Note S1. Bayesian estimation for source parameters

Based on the probability model in the inverse problem, the Bayesian method quantify our state of knowledge by explicitly addressing uncertainties that arise from incomplete information or model inaccuracies during observations, which can attribute a posterior plausibility to each model within a set of constructed models[1,2]. The posterior probability distribution describes the plausibility of each member of this model ensemble, thereby providing the solution to our inverse problem[3]. In order to thoroughly search for possible source parameters, we used the Geodetic Bayesian Inversion Software (GBIS) to apply the nonlinear inversion based on the Bayesian estimation[4]. GBIS uses the Okada[5] model in an elastic, homogeneous half-space to describe the rectangular dislocation source with a uniform slip represented by nine source parameters, which include: the length and width of the rectangular source, the depth of the lower edge, the dip angle with respect to the horizontal, the strike with respect to the north direction, the horizontal Cartesian coordinate of the midpoint of the lower edge, the strike slip along the strike direction, and the dip slip along the dip direction. For the nonlinear inversion, GBIS employs the Markov chain Monte Carlo (MCMC) method incorporating the Metropolis-Hastings algorithm[6,7], with automatic step size selection to characterize the posterior probability distribution of the aforementioned source parameters. Given that the prior information on model parameters is often unpredictable, a non-informative Jeffreys prior[4,8,9] is used, setting the prior distribution of each model parameter to be independent and uniform over a finite range of possible values. Additionally, GBIS is capable of handling multiple independent geodetic datasets. In this study, we utilized the static GNSS displacements and interferometric synthetic aperture radar (InSAR) data for the Bayesian estimation, and the InSAR data were subsampled to reduce the computational complexity.

To explore all possible fault orientations and kinematics, no constraints were imposed onto the fault strike (varying from 0° to 360°) and dip angles (varying from −90° to +90° with respect to the horizontal) and slip direction. The best-fit solution under the uniform slip mode is shown in Fig. S3 and Table S3. The optimal values of the source parameters correlate well with the peaks of the Bayesian estimation. Based on the 95% confidence interval derived from the nonlinear inversion, the GNSS and InSAR data can be explained by a rectangular fault with a length of 51.21–51.80 km, a width of 44.86–45.86 km, striking at 25.27°–25.44° and steeply dipping at 72.68°–72.94°. The fault slip is dominated by a dip-slip component of 1.31–1.33 m, combined by a minor left-lateral strike-slip component of 0.02–0.03 m. Assuming a shear modulus of 6.6×10^4 MPa at the average depth of the fault, the corresponding geodetic moment magnitude M_w is 7.47, slightly larger than those of the released moment tensor solutions (Table S1). Additionally, the fault is located to the NE of the epicenter determined by the U.S. Geological Survey-National Earthquake Information Center (USGS-NEIC), suggesting that the major energy release inferred from the geodetic data is only concentrated on a single side of the epicenter.

Note S2. Backprojection Imaging Analysis

The backprojection imaging method is a powerful tool that can reveal the spatiotemporal migration of rupture radiators using high-frequency teleseismic *P* waves with minimal assumptions. It is therefore extensively used to delineate the features of the rupture complexity, such as rupture velocity, direction, extent, and duration[10-13]. The multiple signal classification (MUSIC) array processing technique, which has been successfully applied in the backprojection imaging[14,15], can distinguish closely spaced simultaneous radiators, thus providing better resolution compared with conventional approaches. To effectively reconstruct the spatiotemporal evolution of high-frequency radiators for the 2024 Hualien earthquake, we employed the MUSIC backprojection method to analyze the waveform traces from three regional seismic arrays with epicentral distances ranging from 30° to 90° to enhance the resolution, including 101 traces from the Alaska array, 235 traces from the Europe array, and 48 traces from the Australia array (Fig. S8). Before the backprojection imaging, the waveforms were band-pass filtered between 0.5 and 2.0 Hz and aligned

relative to a reference trace through cross-correlation. Given the limited depth resolution of the backprojection imaging, the grid search for high-frequency radiators was exclusively conducted on a two-dimensional horizontal plane at a fixed depth. This plane is centered on the epicenter of 23.835°N, 121.598°E, at a depth of 40.0 km determined by the USGS-NEIC, and further divided into grids with an interval of 0.01° along both latitude and longitude. Combining the expected differential slowness of each potential subevent on the gridded horizontal plane, the *P*-wave trains of 10 s long from the first *P*-wave arrival were stacked at every second during 30 s. This procedure determines the most probable location of the high-frequency radiator by identifying the extreme value of the coherent energy release after the waveform stacking.

Compared to the utilization of a single seismic array, the backprojection imaging combining three arrays allows us to better examine the spatiotemporal migration of high-frequency radiators. Based on the common features reflected in the imaging results of the three arrays, we find that more high-frequency radiators are concentrated around the epicenter or to its north, indicating that more seismic energy is predominantly released to the north of the epicenter, while the few radiators to the south side are probably attributed to the residual southward rupture or the swimming effect related to the array azimuth (Fig. S8). Therefore, the 2024 Hualien earthquake is more likely to be a unilateral rupture that mainly extends toward the north, which is close to the Bayesian estimation. To better identify the rupture propagating velocity, we projected the high-frequency radiators derived from the backprojection of three arrays onto the optimal fault strike of 25.4° estimated by the Bayesian inference. As shown in Fig. S9, more high-frequency radiators are projected to the north of the epicenter, and their more convergent distribution also facilitates the identification of the rupture propagation, reflecting an average rupture velocity between 2.0 and 3.0 km/s. In the joint finite-fault inversion combining seismic and geodetic observations, we conducted a systematic grid search to obtain the optimal rupture velocity. The results of the backprojection with multiple arrays also provides an effective reference for selecting the rupture velocity. The rupture velocity was ultimately selected to be 2.5 km/s for the joint finite-fault inversion, which also matches well with the rupture process reflected by the backprojected high-frequency radiators from multiple arrays.

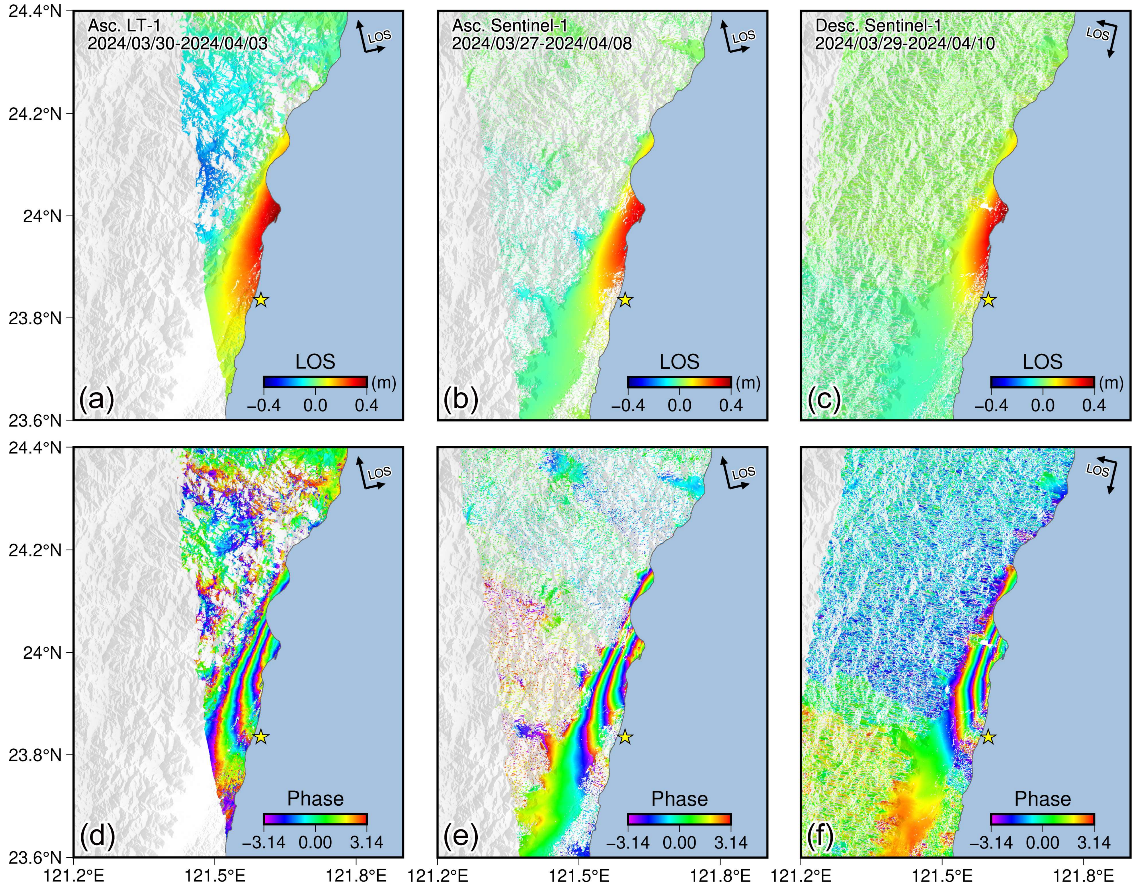

Fig. S1. (a–c) DInSAR LOS unwrapped displacements and (d–f) wrapped interferograms in the ascending orbit of the LT-1 satellite and in the ascending and descending orbits of the Sentinel-1 satellite. The maps were generated by the Generic Mapping Tools 6.3.0 (GMT 6.3.0, https://www.generic-mapping-tools.org).

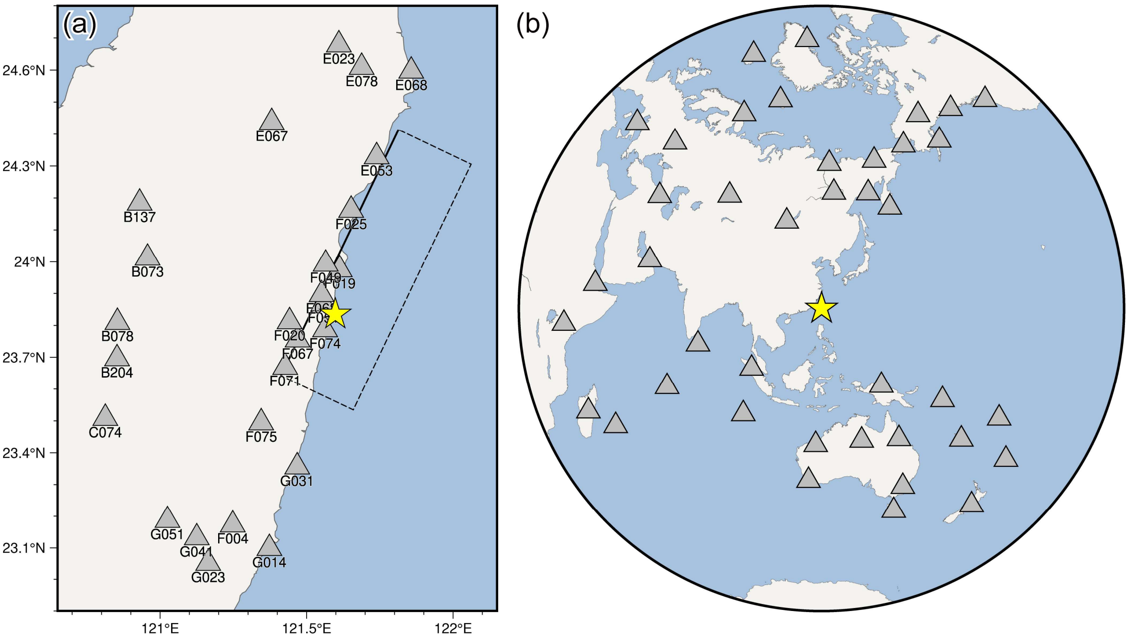

Fig. S2. (a) Distribution of the strong-motion stations with station names used in the joint finite-fault inversion. The ground projection of our preferred finite-fault model for the 2024 Hualien earthquake are shown within the black frame in the map view, with the solid line indicating the top edge. (b) Distribution of the teleseismic stations used in the joint finite-fault inversion. The yellow star represents the epicenter. The maps were generated by the Generic Mapping Tools 6.3.0 (GMT 6.3.0, https://www.generic-mapping-tools.org).

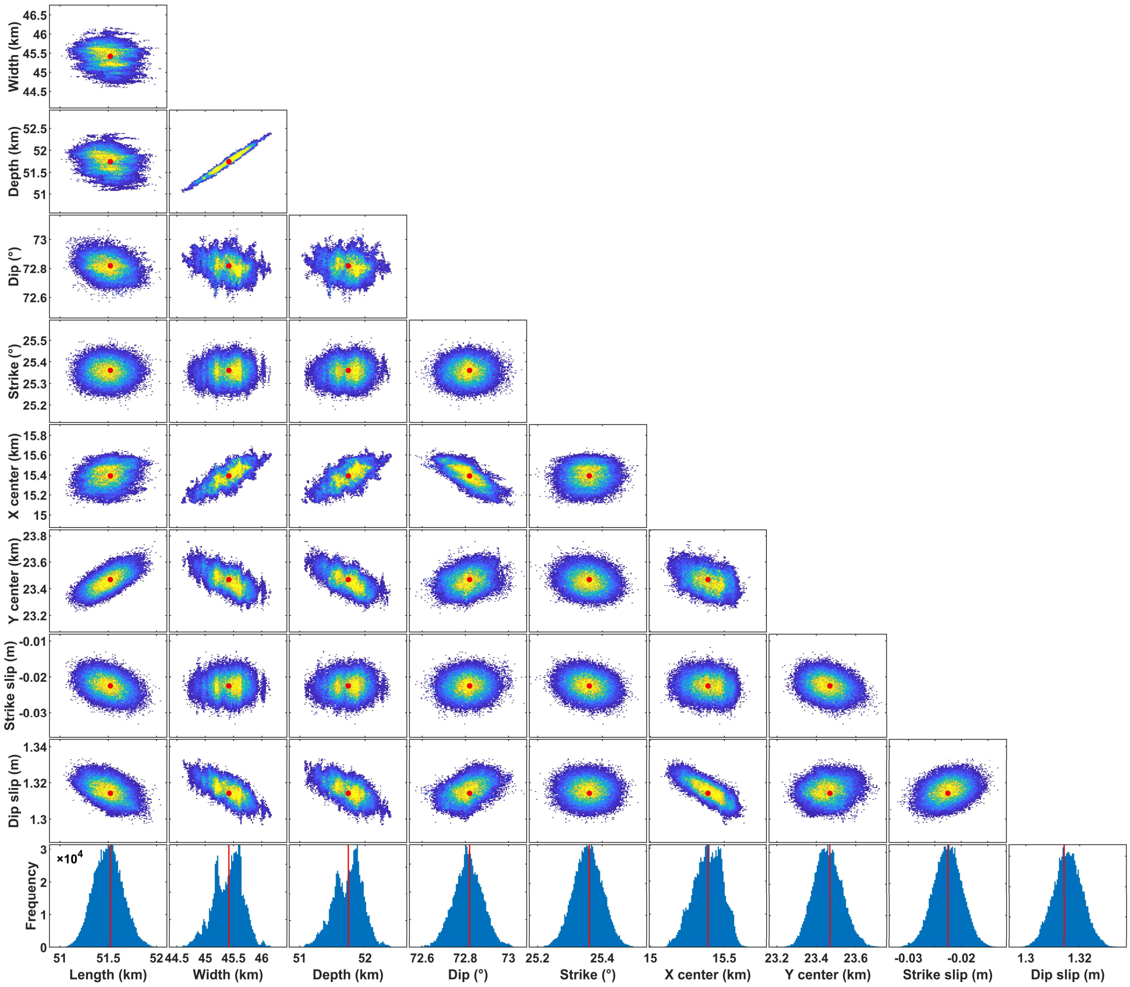

Fig. S3. Marginal posterior probability distributions of the source parameters derived from the Bayesian estimation. Red lines represent the best-fit values of parameters. Scatter plots are contoured according to frequency, with cold colors for low frequency and warm colors for high frequency.

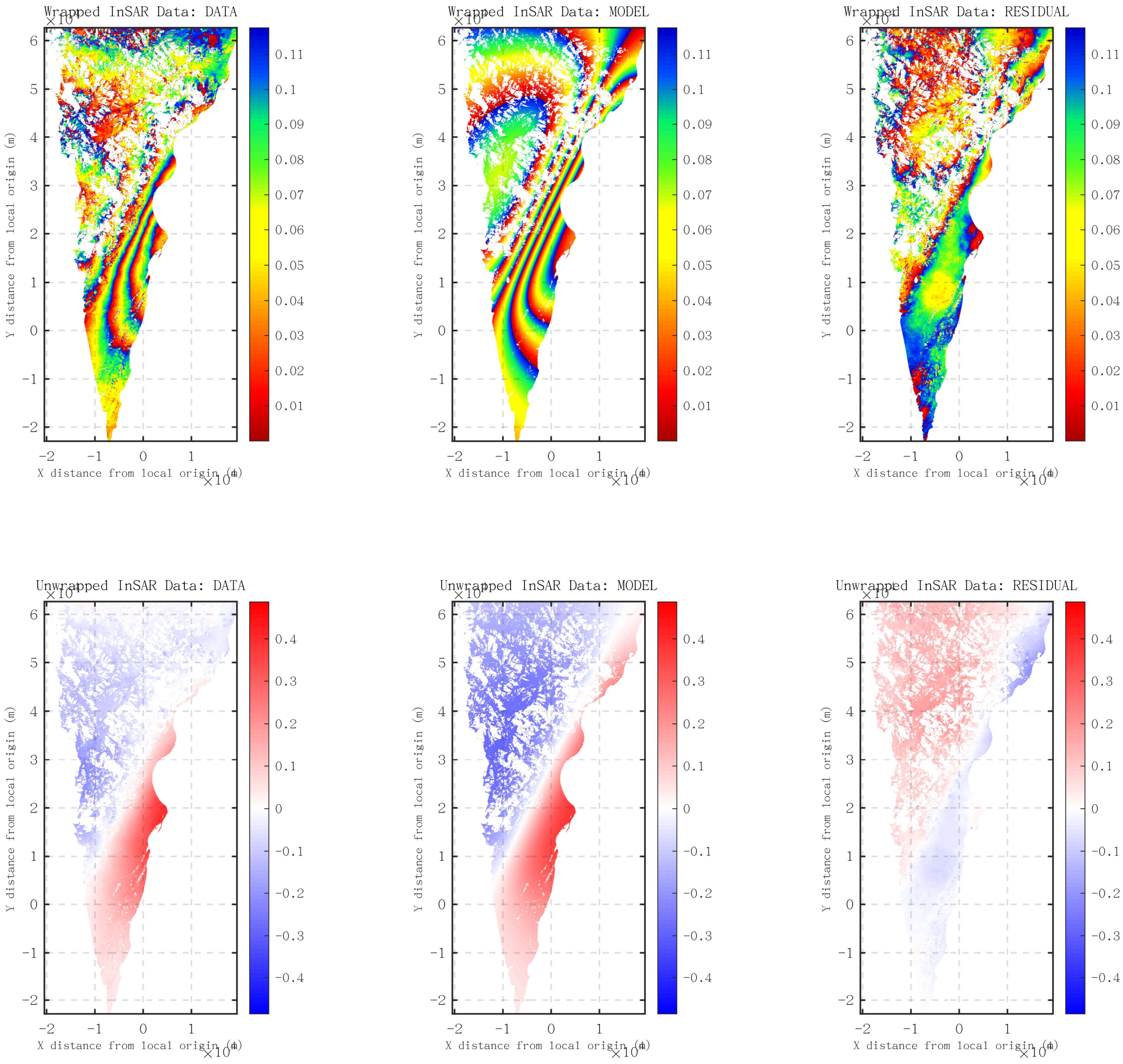

Fig. S4. Unwrapped LOS displacements and wrapped interferograms of the observed, modeled and residual in the ascending orbit of the LT-1 satellite derived from the Bayesian estimation.

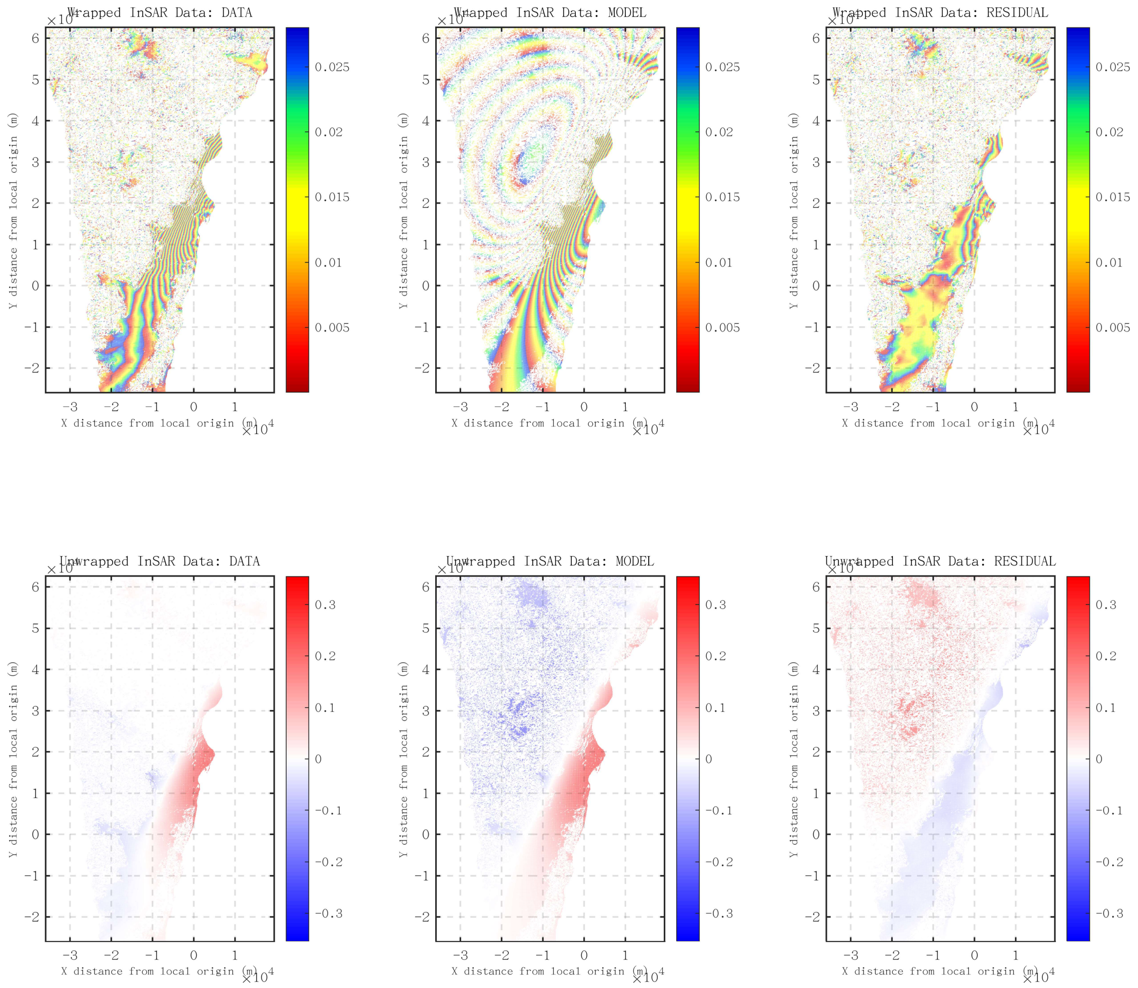

Fig. S5. Unwrapped LOS displacements and wrapped interferograms of the observed, modeled and residual in the ascending orbit of the Sentinel-1 satellite derived from the Bayesian estimation.

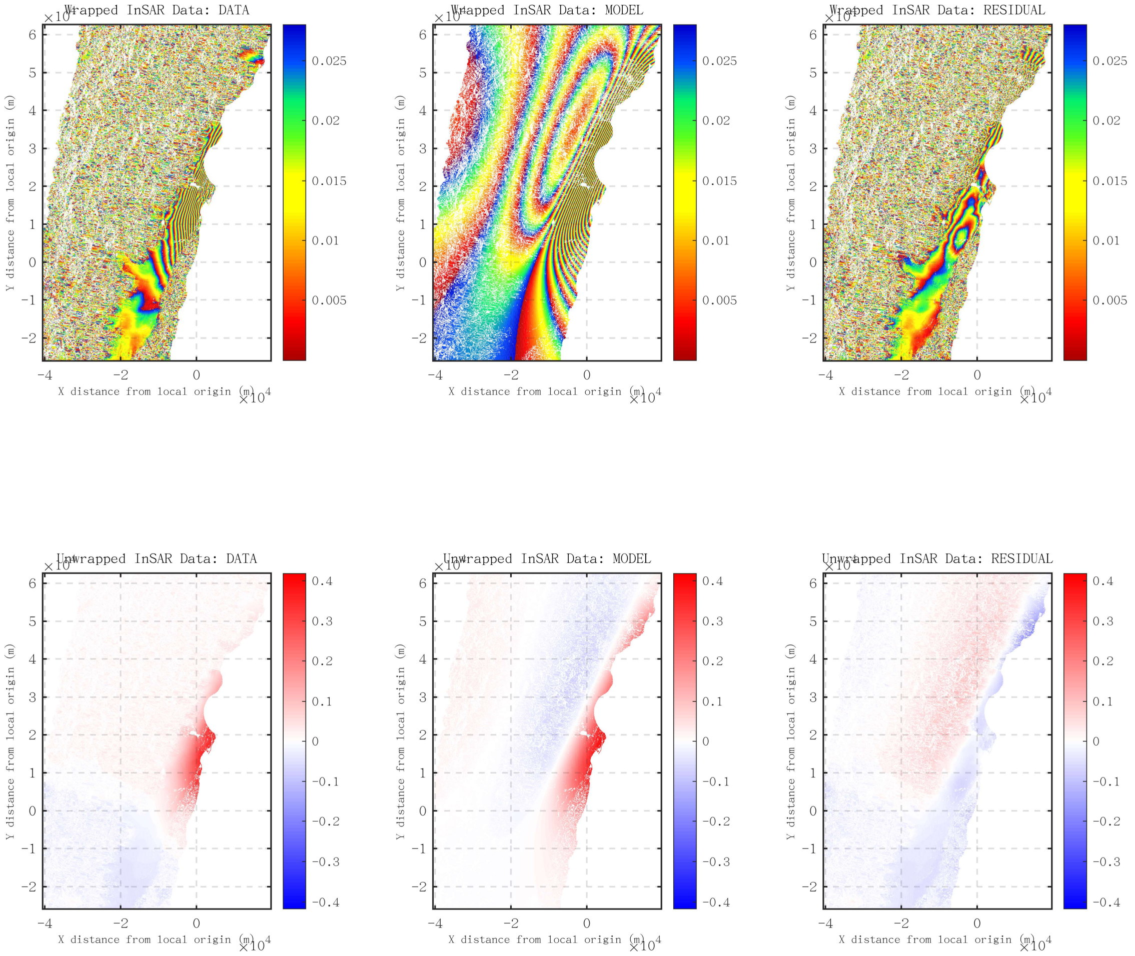

Fig. S6. Unwrapped LOS displacements and wrapped interferograms of the observed, modeled and residual in the descending orbit of the Sentinel-1 satellite derived from the Bayesian estimation.

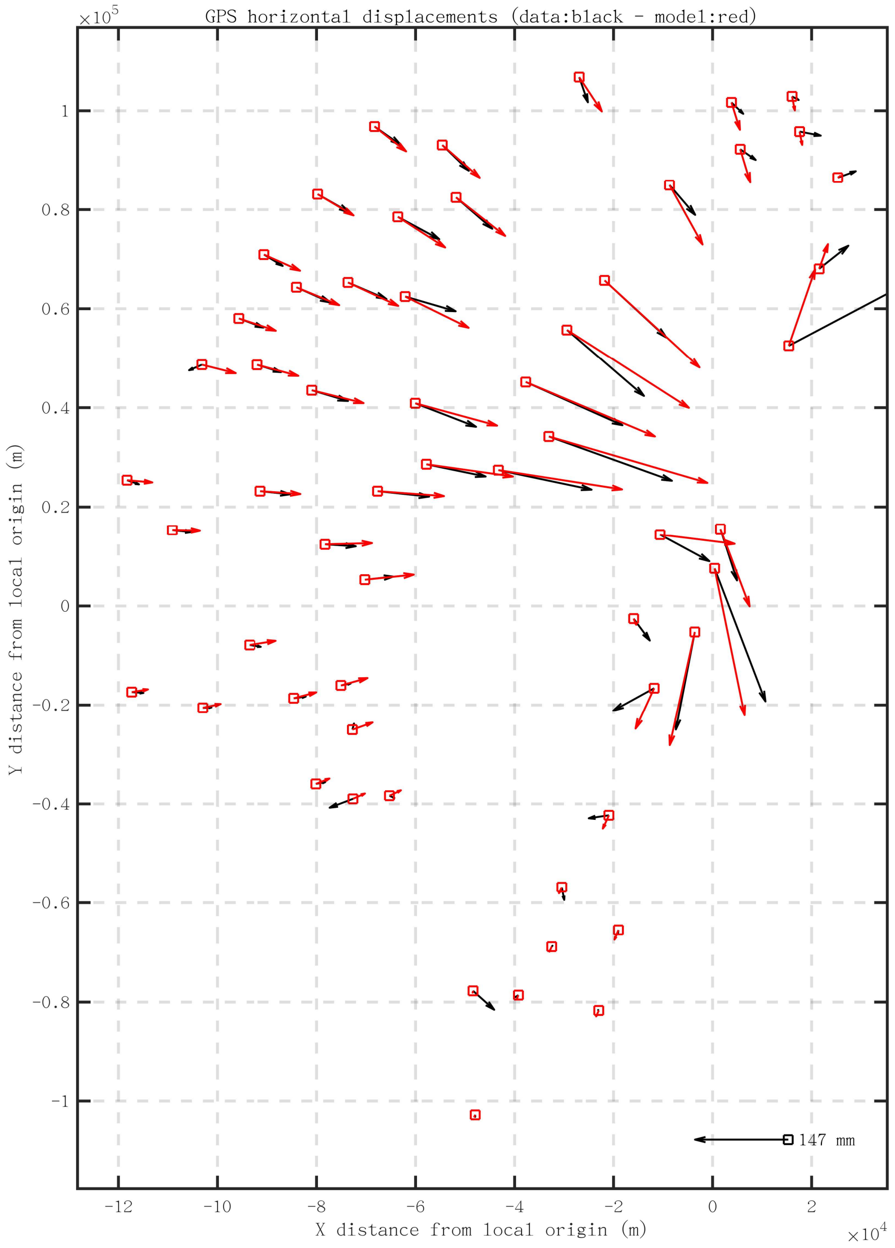

Fig. S7. Comparison between the observed and synthetic GNSS displacements obtained from the Bayesian estimation.

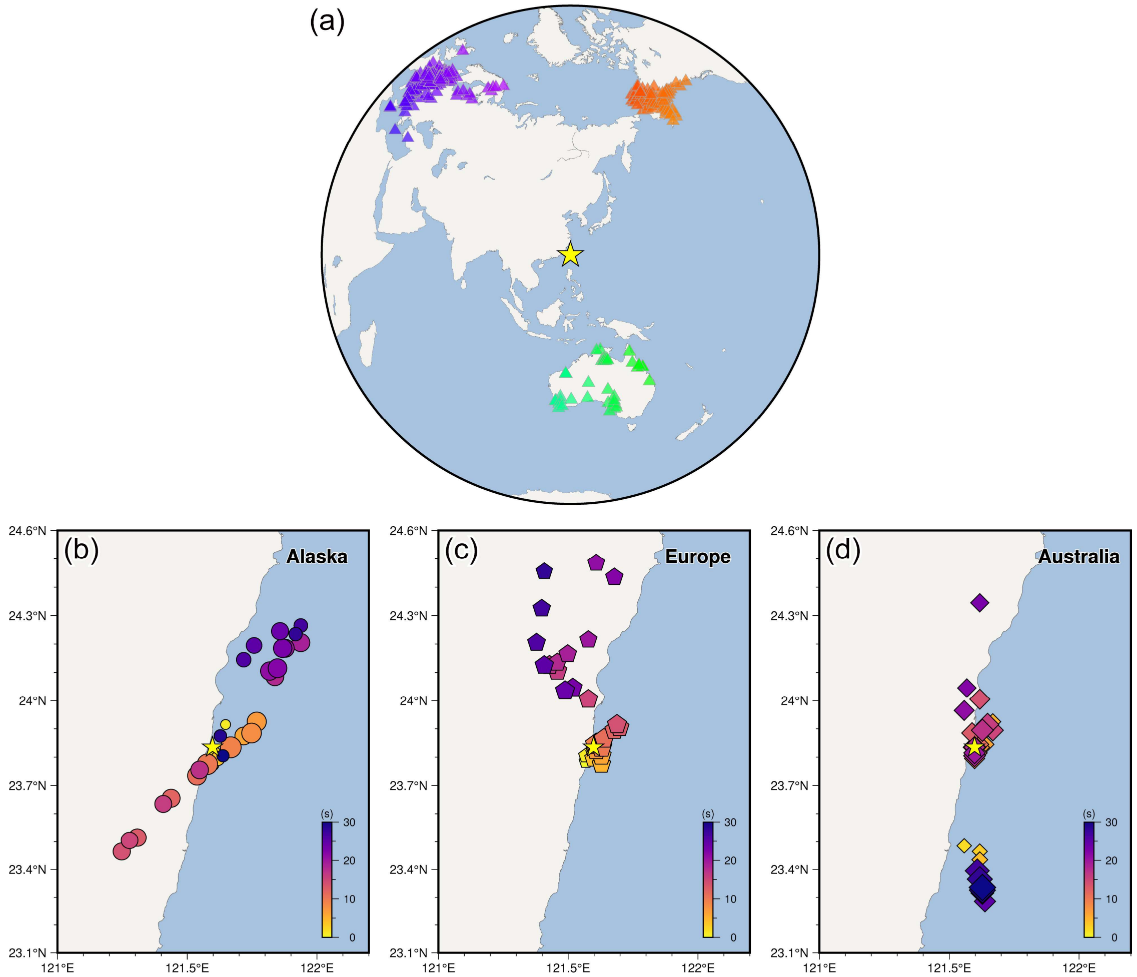

Fig. S8. (a) Distribution of the seismic stations (colored triangles) of the Alaska, Europe and Australia arrays used in the backprojection imaging analysis. (b–d) Spatiotemporal migration of the high-frequency radiators derived from the backprojection imaging using (b) the Alaska array, (c) the Europe array, and (d) the Australia array. The maps were generated by the Generic Mapping Tools 6.3.0 (GMT 6.3.0, https://www.generic-mapping-tools.org).

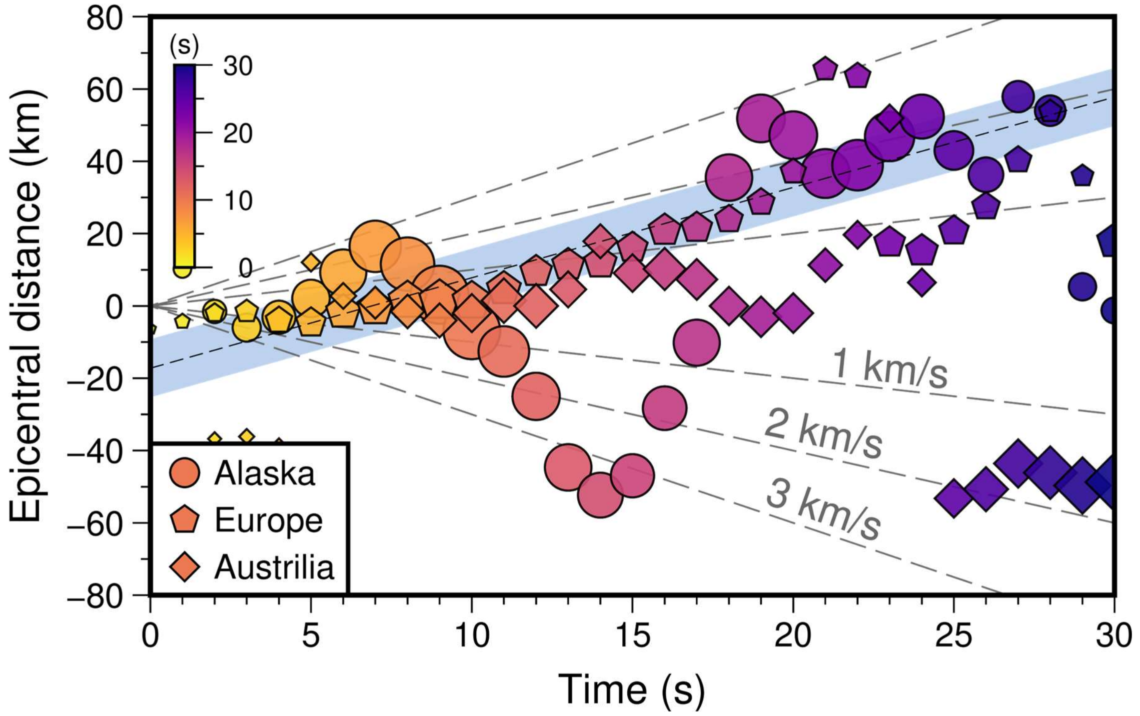

Fig. S9. Distance of the backprojected high-frequency radiators away from the epicenter versus the rupture time. Reference rupture velocities of 1 km/s, 2 km/s and 3km/s are indicated by the dashed gray lines. The rupture velocity of 2.5 km/s, which is preferred for the joint finite-fault inversion, is indicated by the blue sheet.

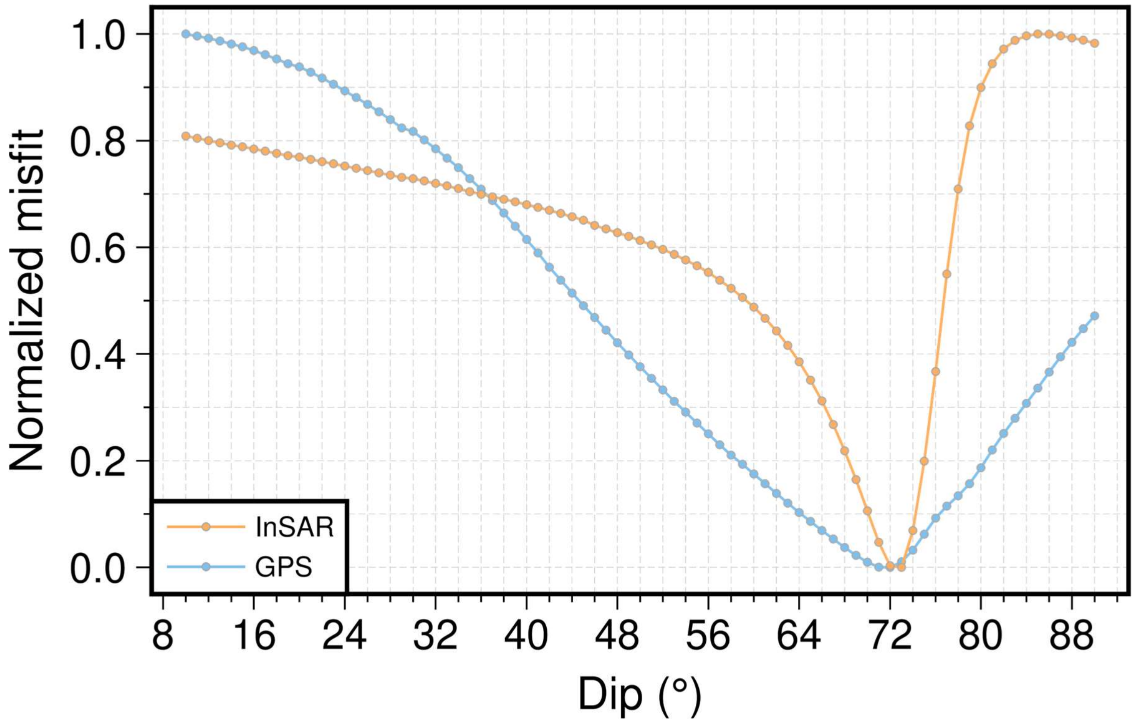

Fig. S10. Trade-off curves between the fault dip and the normalized misfits of the InSAR and static GNSS displacements in the finite-fault inversion trials using the geodetic data.

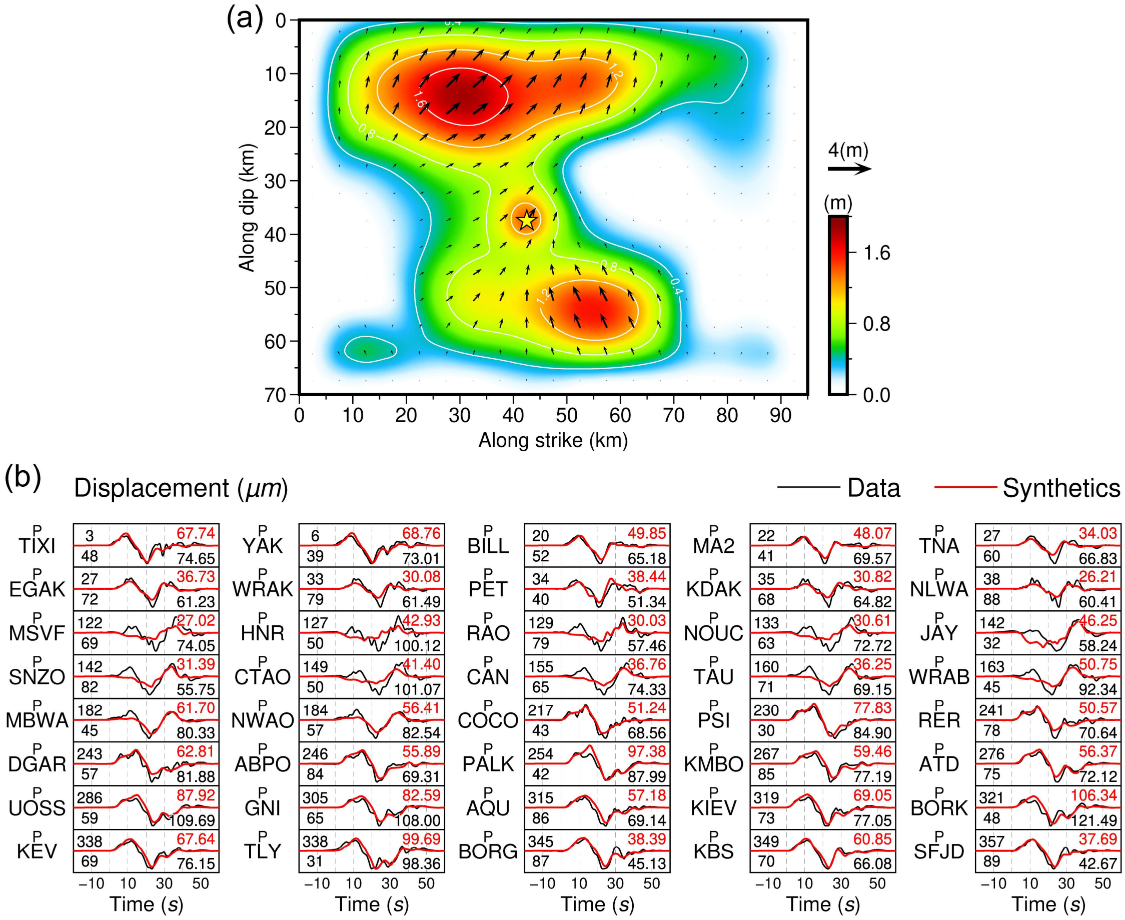

Fig. S11. (a) Slip distribution of the planar fault model with the fault dip of 72.8° derived from the finite-fault inversion using the teleseismic *P* waves. (b) Comparison between the observed teleseismic *P* waves (displacement) and the synthetic waveforms obtained from the finite-fault inversion. The maximum amplitudes of the observed (black font) and synthetic waveforms (red font) are shown to the right of each waveform, in micrometers. The azimuth and epicentral distance in degrees are shown at the beginning of each record with the azimuth on top.

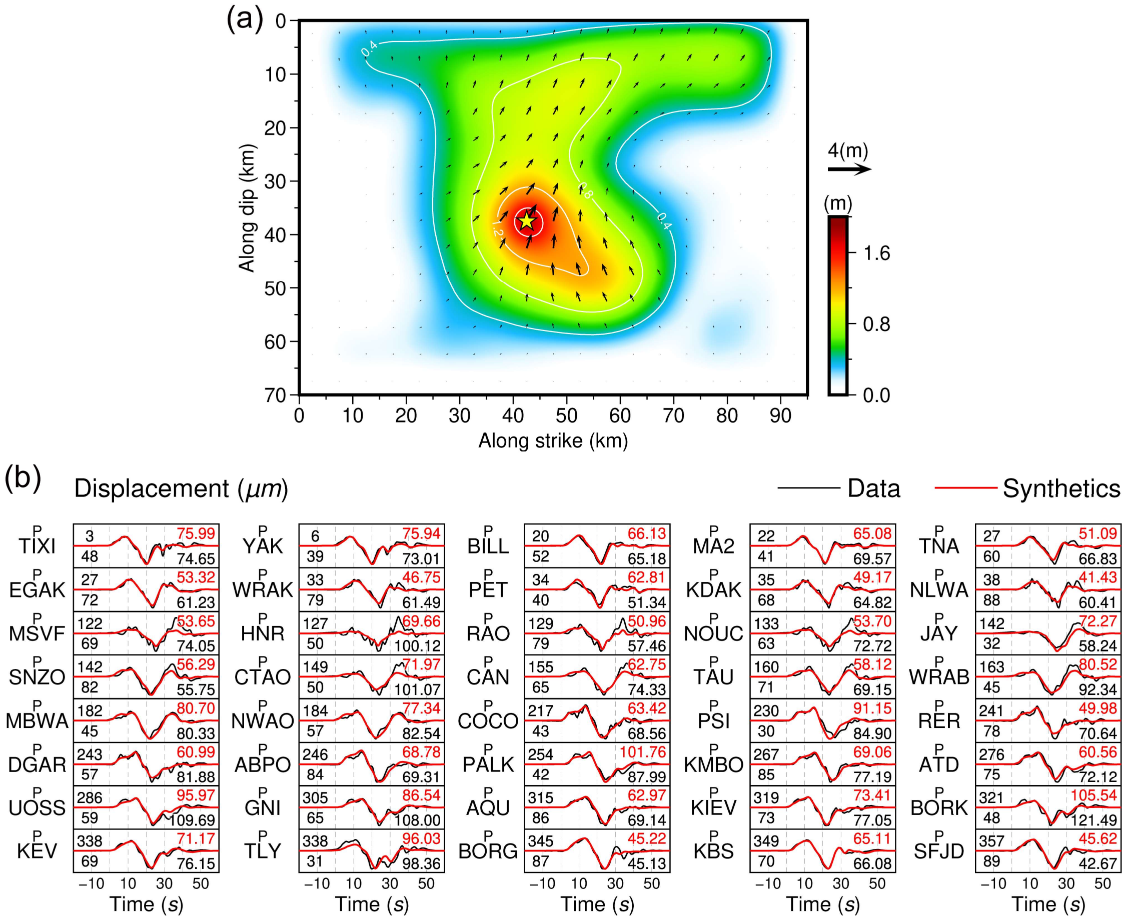

Fig. S12. (a) Slip distribution of the planar fault model with the fault dip of 54.0° derived from the finite-fault inversion using the teleseismic *P* waves. (b) Comparison between the observed teleseismic *P* waves (displacement) and the synthetic waveforms obtained from the finite-fault inversion. The maximum amplitudes of the observed (black font) and synthetic waveforms (red font) are shown to the right of each waveform, in micrometers. The azimuth and epicentral distance in degrees are shown at the beginning of each record with the azimuth on top.

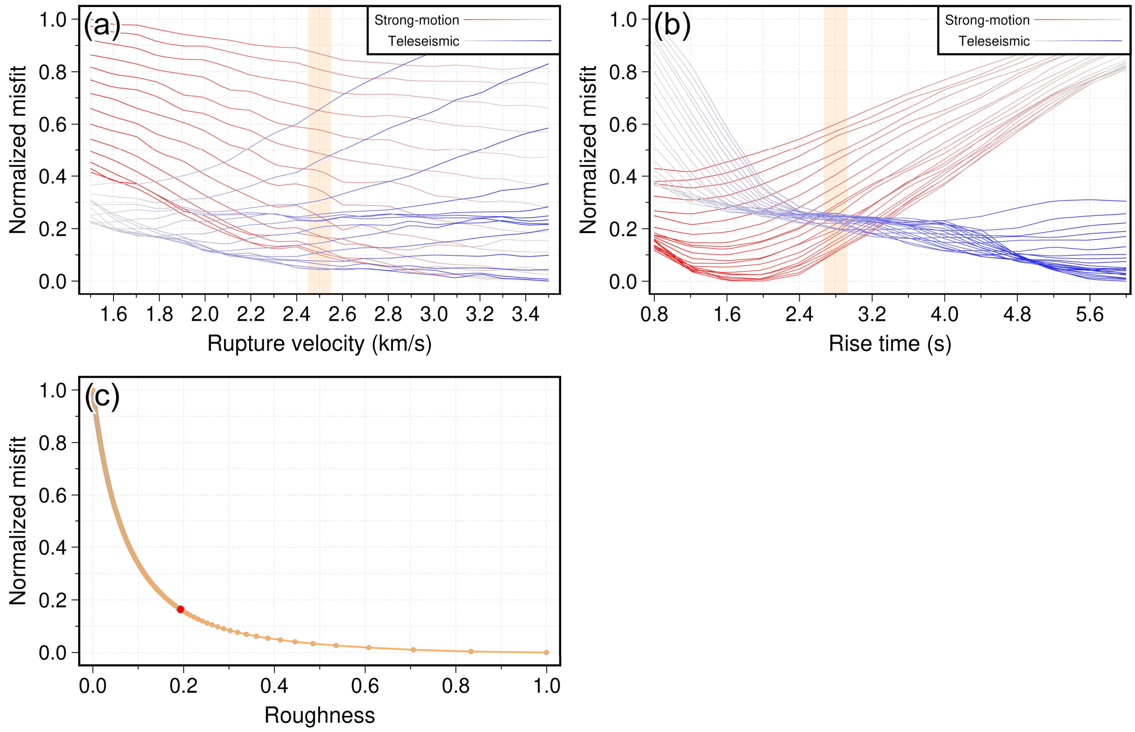

Fig. S13. (a) Trade-off curves between the normalized misfits of strong-motion and teleseismic waveforms and the rupture velocity. Each curve corresponds to a candidate of the testing rise times of time-window. (b) Trade-off curves between the normalized misfits of strong-motion and teleseismic waveforms and the rise time of time-window. Each curve corresponds to a candidate of the testing rupture velocities. (c) Trade-off curve between the model roughness and the normalized misfit tested with different smoothing factors for the joint finite-fault inversion. The smoothing factors from 0.00005 to 0.01 with a step of 0.00005 are tested. The red solid circle indicates that the preferred value of the smoothing factor is 0.0012.

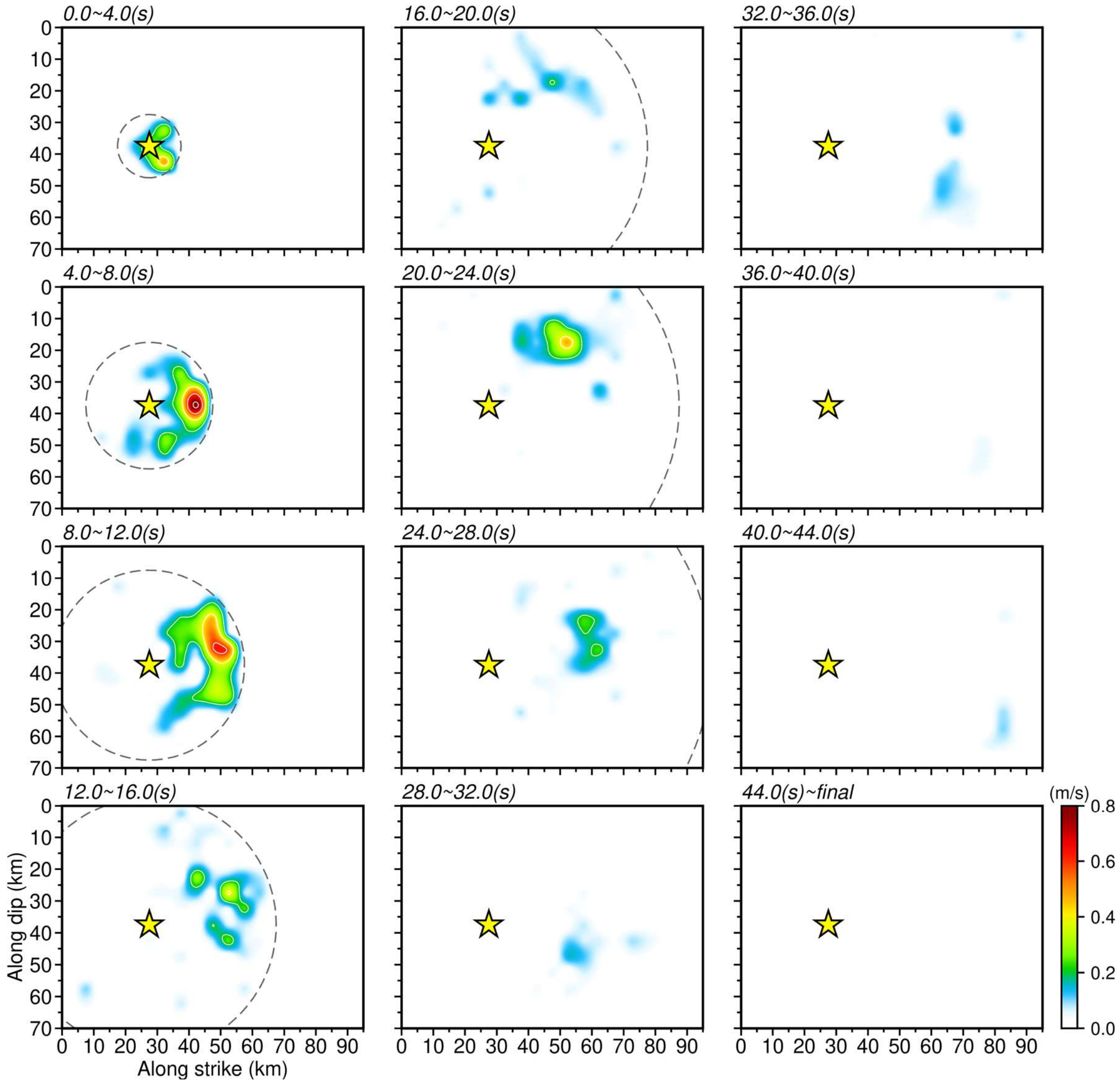

Fig. S14. Source rupture process represented by the slip rate evolution of our preferred fault model. The contour interval is 0.2 m/s, and the yellow star indicates the epicenter. The gray dashed line represents the rupture front corresponding to a rupture velocity of 2.5 km/s.

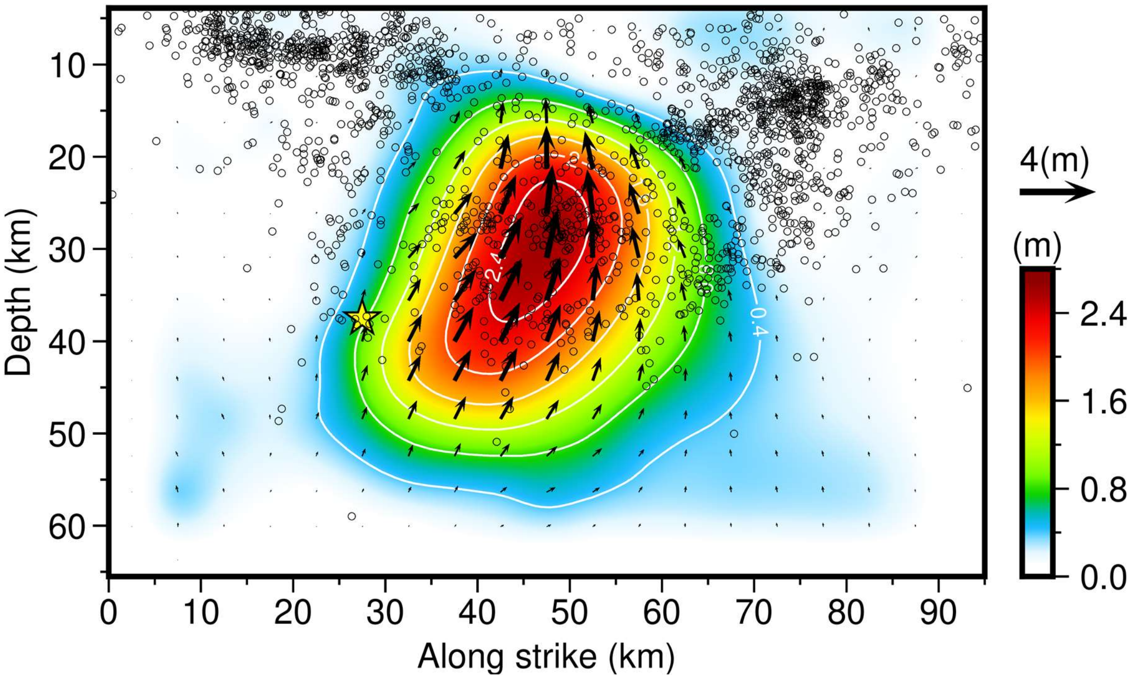

Fig. S15. The aftershock distribution projected into the fault plane of the 2024 Hualien earthquake, which are indicated by black circles. The aftershocks of the seismic swarm on 22 April 2024 are excluded. The slip distribution with slip contours in white is depicted as the background color.

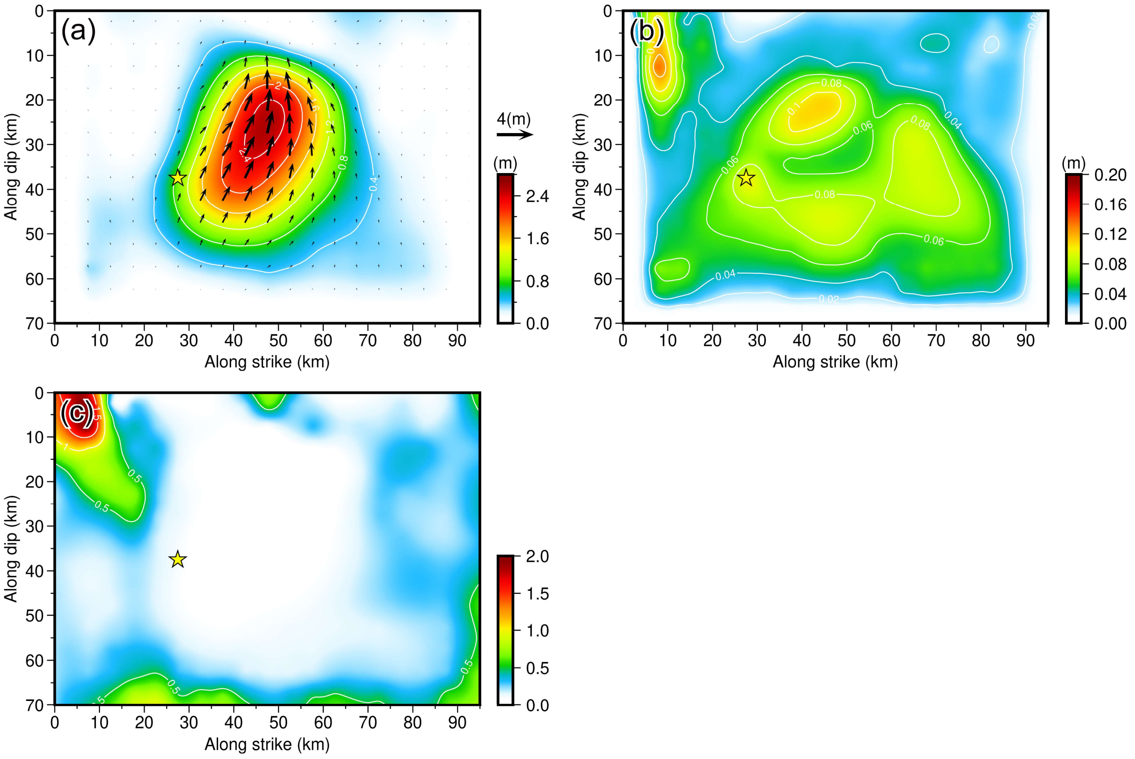

Fig. S16. Distributions of (a) the mean slip, (b) the standard deviation and (c) the coefficient of variation in the Jackknife testing.

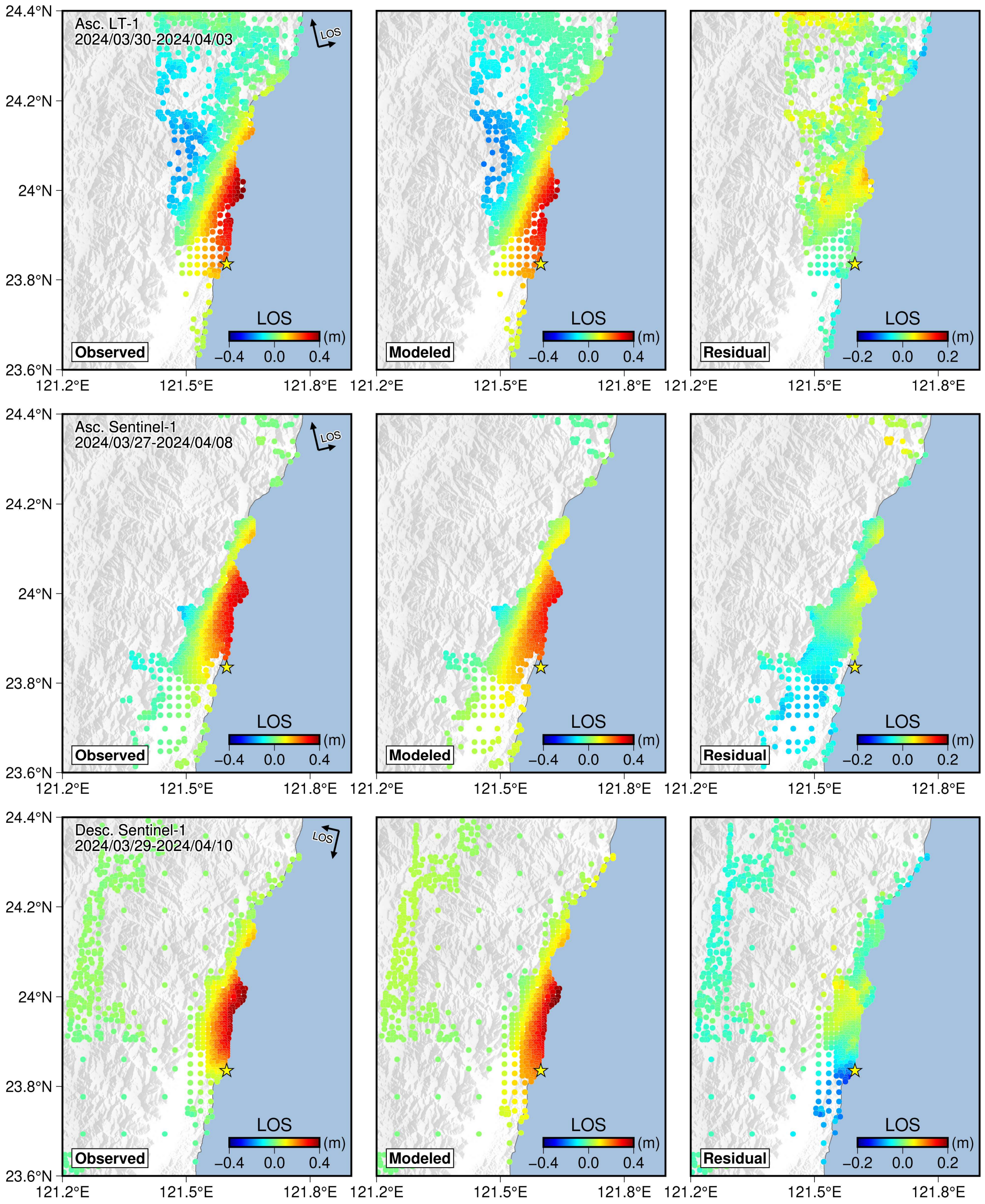

Fig. S17. Unwrapped LOS displacements of the observed, modeled and residual in the ascending orbit of the LT-1 satellite and in the ascending and descending orbits of the Sentinel-1 satellite derived from the joint finite-fault inversion. The maps were generated by the Generic Mapping Tools 6.3.0 (GMT 6.3.0, https://www.generic-mapping-tools.org).

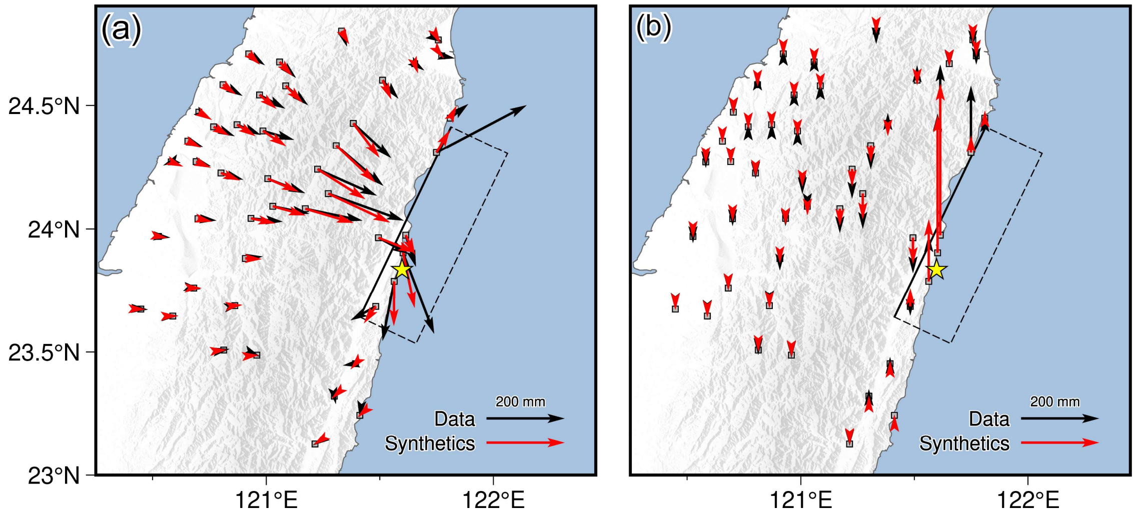

Fig. S18. Comparison between the observed (a) horizontal and (b) vertical components of the static GNSS displacements (black arrow) and the synthetic ones (red arrows) obtained from the joint finite-fault inversion. The maps were generated by the Generic Mapping Tools 6.3.0 (GMT 6.3.0, https://www.generic-mapping-tools.org).

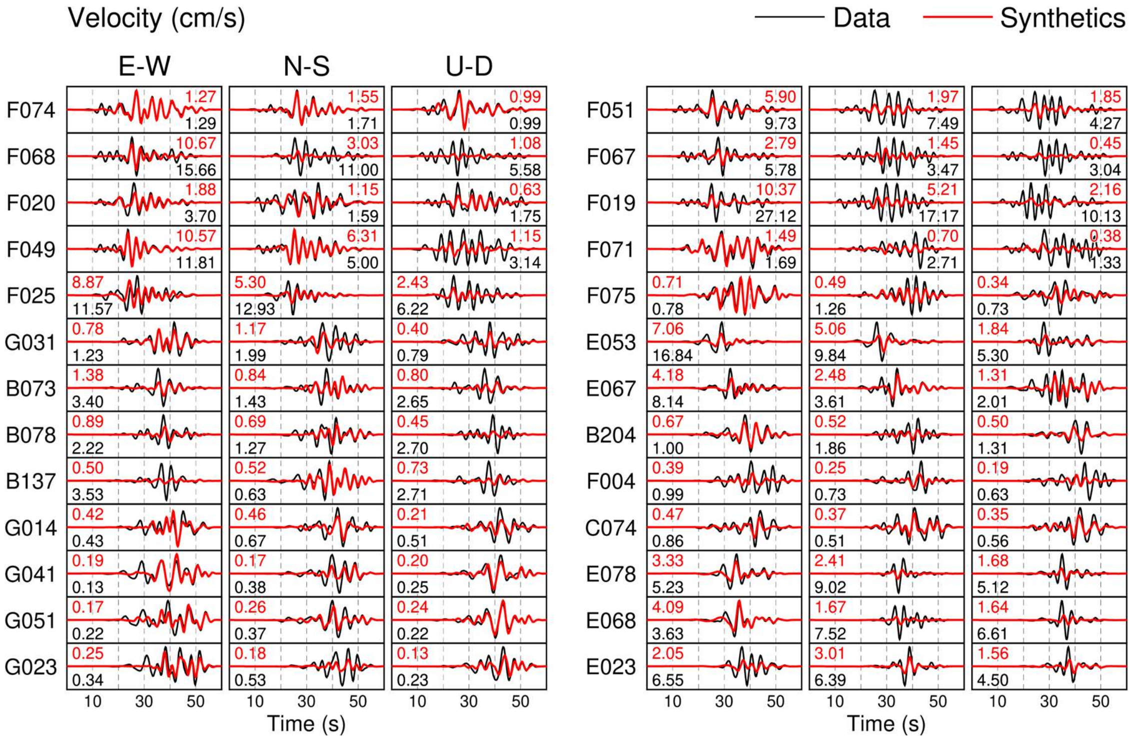

Fig. S19. Comparison between the observed strong-motion seismograms (velocity) and the synthetic waveforms obtained from the joint finite-fault inversion. The maximum amplitudes of the observed (black font) and synthetic waveforms (red font) are shown to the left or right of each waveform, in centimeters per second.

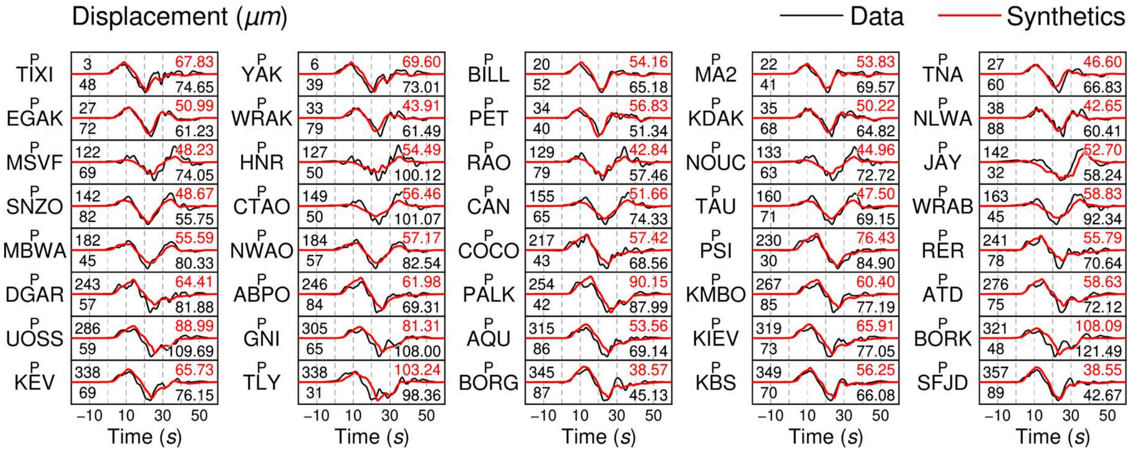

Fig. S20. (b) Comparison between the observed teleseismic *P* waves (displacement) and the synthetic waveforms obtained from the joint finite-fault inversion. The maximum amplitudes of the observed (black font) and synthetic waveforms (red font) are shown to the right of each waveform, in micrometers. The azimuth and epicentral distance in degrees are shown at the beginning of each record with the azimuth on top.

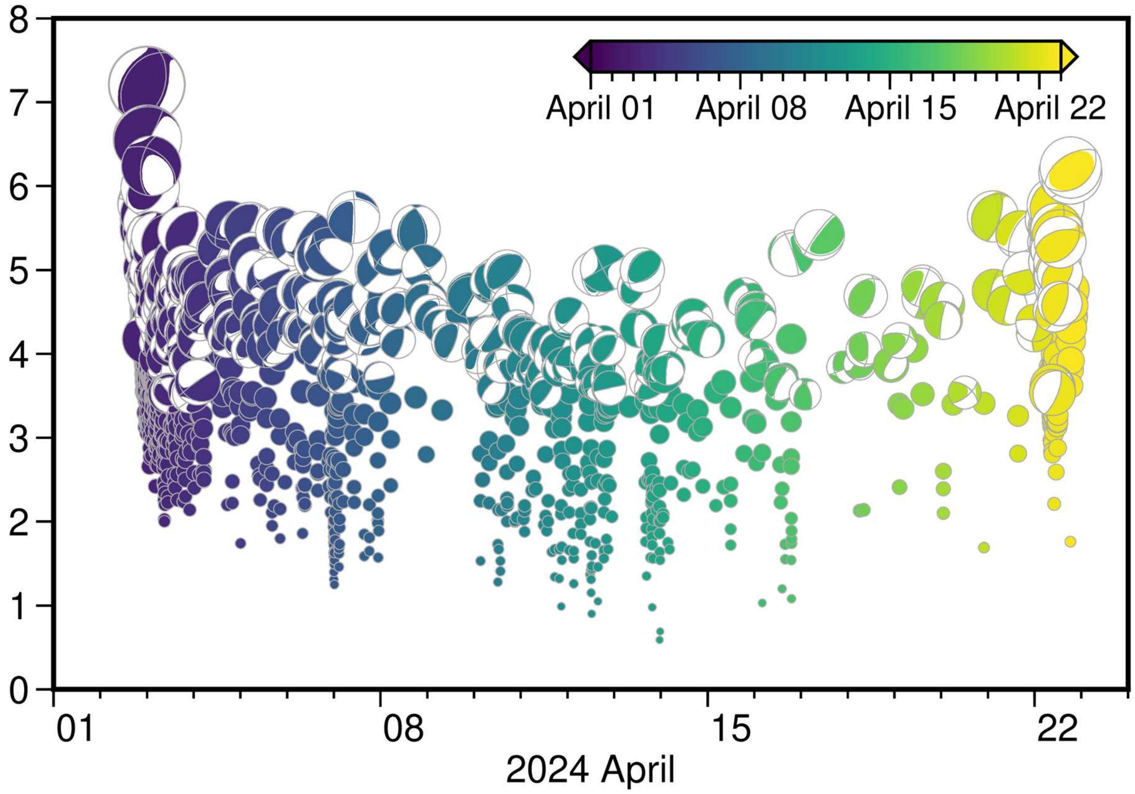

Fig. S21. Time series of the seismicity from the mainshock on 2 April 2 2024 to 22 April 2024, in which earthquakes are colored by time. The event catalog is collected from the Taiwan Seismological and Geophysical Data Management System (GDMS). And the moment tensors of some events are collected from the Broadband Array in Taiwan for Seismology (BATS) solutions.

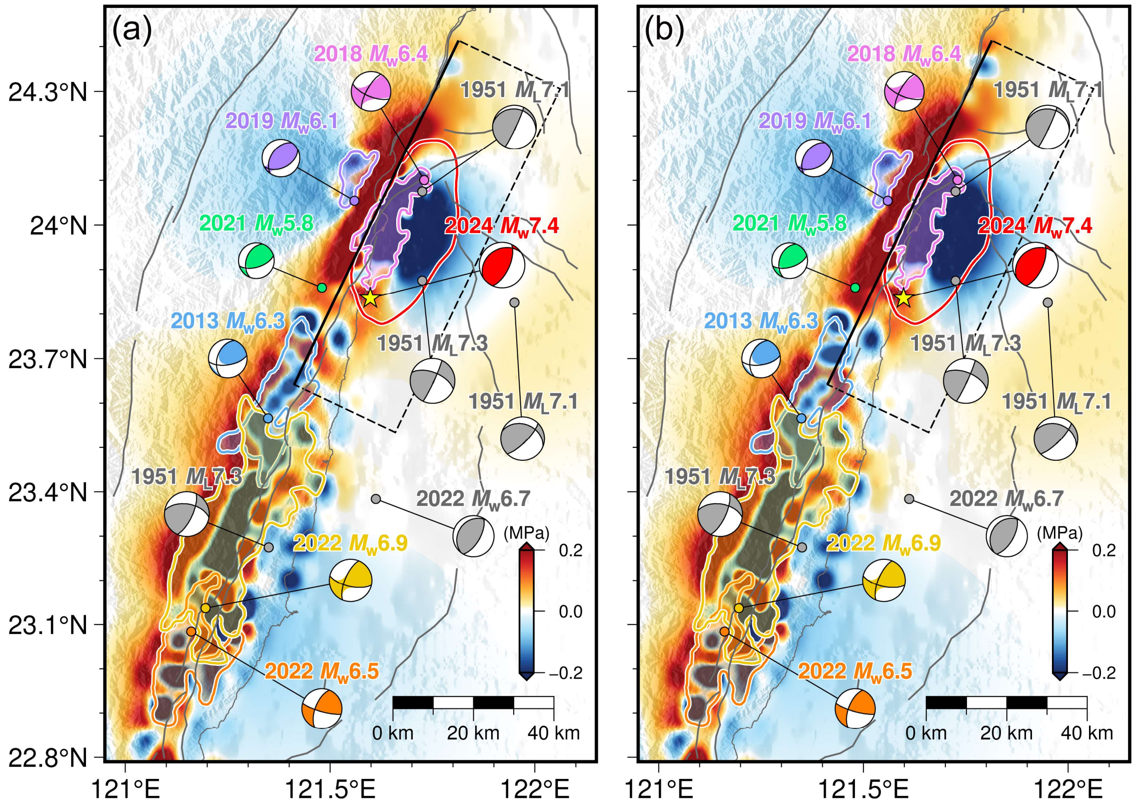

Fig. S22. Regional field of the cumulative CFS changes induced by the representative historical major earthquakes since 2013, including the 2024 Hualien earthquake. For the 2024 event, the slip uncertainties at the 95% confidence level indicated by the Jackknife test were taken into account. The standard deviation of 1.96 times was (a) subtracted from and (b) added to the mean slip distribution, respectively. The maps were generated by the Generic Mapping Tools 6.3.0 (GMT 6.3.0, https://www.generic-mapping-tools.org).

Table S1. Moment tensor solutions of the 2024 Hualien earthquake.

Source	M_w	Centroid depth (km)	Nodal plane #1			Nodal plane #2		
			Strike (°)	Dip (°)	Rake (°)	Strike (°)	Dip (°)	Rake (°)
USGS-NEIC	7.37	35.5	19	54	73	227	40	112
GCMT	7.40	33.7	17	57	70	230	38	117
BATS	7.30	35.0	16	51	79	213	40	103

Table S2. LT-1 and Sentinel-1 SAR acquisitions used in this study.

Satellite	Track number	Preseismic acquisition date	Postseismic acquisition date
LT-1	Ascending	2024/03/30	2024/04/03
Sentinel-1	Ascending 69	2024/03/27	2024/04/08
Sentinel-1	Descending 105	2024/03/29	2024/04/10

Table S3. Priors and results of the Bayesian estimation for the 2024 Hualien Earthquake.

	Length (m)	Width (m)	Depth (m)	Dip (°)	Strike (°)	X center (m)	Y center (m)	Strike slip[a] (m)	Dip slip[b] (m)
**Lower**	10000	10000	1000	−90.0	0.0	−50000	−50000	-5.0	-5.0
**Upper**	100000	100000	100000	90.0	360.0	50000	50000	5.0	5.0
**Optimal**	51522	45418	51743	72.80	25.36	15390	23459	−0.022	1.314
**2.5%**	51213	44859	51275	72.68	25.27	15189	23321	−0.028	1.305
**97.5%**	51798	45856	52135	72.94	25.44	15562	23611	−0.017	1.326

[a]Strike slip is positive if right-lateral and negative if left-lateral. [b]Dip slip is positive for thrust faulting and negative for normal faulting.

References

1. Jaynes, E. T. & Bretthorst, G. L. *Probability Theory: The Logic of Science*. (Cambridge University Press, 2003).
2. Beck, J. L. Bayesian system identification based on probability logic. *Structural Control & Health Monitoring* **17**, 825-847, doi:10.1002/stc.424 (2010).
3. Minson, S. E., Simons, M. & Beck, J. L. Bayesian inversion for finite fault earthquake source models I-theory and algorithm. *Geophysical Journal International* **194**, 1701-1726, doi:10.1093/gji/ggt180 (2013).
4. Bagnardi, M. & Hooper, A. Inversion of Surface Deformation Data for Rapid Estimates of Source Parameters and Uncertainties: A Bayesian Approach. *Geochemistry Geophysics Geosystems* **19**, 2194-2211, doi:10.1029/2018gc007585 (2018).
5. Okada, Y. Surface Deformation Due to Shear and Tensile Faults in a Half-Space. *Bulletin of the Seismological Society of America* **75**, 1135-1154 (1985).
6. Hastings, W. K. Monte-Carlo Sampling Methods Using Markov Chains and Their Applications. *Biometrika* **57**, 97-&, doi:10.1093/biomet/57.1.97 (1970).
7. Mosegaard, K. & Tarantola, A. Monte-Carlo Sampling of Solutions to Inverse Problems. *Journal of Geophysical Research-Solid Earth* **100**, 12431-12447, doi:10.1029/94jb03097 (1995).
8. Jeffreys, H. *Theory of probability*. 3rd edn, (Clarendon Press, 1983).
9. Ulrych, T. J., Sacchi, M. D. & Woodbury, A. A Bayes tour of inversion: A tutorial. *Geophysics* **66**, 55-69, doi:10.1190/1.1444923 (2001).
10. Ishii, M., Shearer, P. M., Houston, H. & Vidale, J. E. Extent, duration and speed of the 2004 Sumatra-Andaman earthquake imaged by the Hi-Net array. *Nature* **435**, 933-936, doi:10.1038/nature03675 (2005).
11. Kruger, F. & Ohrnberger, M. Tracking the rupture of the Mw=9.3 Sumatra earthquake over 1,150 km at teleseismic distance. *Nature* **435**, 937-939, doi:10.1038/nature03696 (2005).
12. Yao, H. J., Shearer, P. M. & Gerstoft, P. Subevent location and rupture imaging using iterative backprojection for the 2011 Tohoku Mw 9.0 earthquake. *Geophysical Journal International* **190**, 1152-1168, doi:10.1111/j.1365-246X.2012.05541.x (2012).
13. Kiser, E. & Ishii, M. Back-Projection Imaging of Earthquakes. *Annual Review of Earth and Planetary Sciences* **45**, 271-299, doi:10.1146/annurev-earth-063016-015801 (2017).
14. Meng, L. S., Inbal, A. & Ampuero, J. P. A window into the complexity of the dynamic rupture of the 2011 Mw 9 Tohoku-Oki earthquake. *Geophysical Research Letters* **38**, doi:10.1029/2011gl048118 (2011).
15. Bao, H., Ampuero, J. P., Meng, L. S. *et al.* Early and persistent supershear rupture of the 2018 magnitude 7.5 Palu earthquake. *Nature Geoscience* **12**, 200-205, doi:10.1038/s41561-018-0297-z (2019).
